# Cost-effectiveness of adrenaline for out-of-hospital cardiac arrest

**DOI:** 10.1186/s13054-020-03271-0

**Published:** 2020-09-27

**Authors:** Felix Achana, Stavros Petrou, Jason Madan, Kamran Khan, Chen Ji, Anower Hossain, Ranjit Lall, Anne-Marie Slowther, Charles D. Deakin, Tom Quinn, Jerry P. Nolan, Helen Pocock, Nigel Rees, Michael Smyth, Simon Gates, Dale Gardiner, Gavin D. Perkins, for the PARAMEDIC2 Collaborators, Stavros Petrou, Jason Madan, Kamran Khan, Chen Ji, Anower Hossain, Ranjit Lall, Anne-Marie Slowther, Charles D. Deakin, Tom Quinn, Jerry P. Nolan, Helen Pocock, Nigel Rees, Michael Smyth, Simon Gates, Dale Gardiner, Gavin D. Perkins, Matthew Cooke, Sarah Lamb, Andrew Carson, Ian Jacobs, Ed England, John Black, Nicola Brock, Claire Godfrey, Sarah Taylor, Michelle Thomson, Isabel Rodriguez-Bachiller, Claire King, Marie Stevens, Johanna Lazarus, Helen Werts, Joshua Golding, Rachel Fothergill, Fionna Moore, Alex Boda, Richard Whitfield, Laura Galligan, Rob Lovett, Jennifer Bradley, Lyndsay O’Shea, Mark Docherty, Imogen Gunsen, Gill Price, Andy Rosser, Garry Parcell, Mindy Jhamat, Josh Miller, Jenny Sears Brown, Alice Pretty, Madison Larden, Emma Harris, Jenny Lumley-Holmes, Rhiannon Boldy, Prudence Horwood, Kyee Han, Karl Charlton, Sonia Byers, Gary Shaw, Matt Limmer, Craig Wynne, Michelle Jackson, Emma Bell, Oliver Gupta, Rima Gupta, Charlotte Scomparin, Susie Hennings, Jessica Horton, James Buck, Sarah Rumble, Hayley Johnson, Eva Kritzer, Chockalingham Muthiah, Adrian Willis, Claire Daffern, Louise Clarkson, Felix Achana, Nicola Cashin, Emma Skilton, Malvenia Richmond, Martin Underwood, Natalie Strickland, Sarah Duggan, Scott Regan, Jill Wood, Jon Nicholl, Neil Bayliss, Helen Snooks, Jonathan Benger, Robert Andrews, David Pitcher, William Lee, Matt Wise, Marion Campbell, Jasmeet Soar, Kathy Rowan, Sue Mason

**Affiliations:** 1 Warwick Clinical Trials Unit, Warwick Medical School, The University of Warwick, Coventry, UK; 2 Nuffield Department of Primary Care Health Sciences, University of Oxford, Oxford, UK; 3 Institute of Statistical Research and Training (ISRT), University of Dhaka, Dhaka, Bangladesh; 4 Southampton Respiratory Biomedical Research Unit, National Institute for Health Research, Southampton, UK; 5 Kingston University and St. George’s, University of London, London, UK; 6 Critical Care Unit, Royal United Hospital, Bath, UK; 7 South Central Ambulance Service NHS Foundation Trust, Otterbourne, UK; 8 Welsh Ambulance Services NHS Trust, Swansea, UK; 9 West Midlands Ambulance NHS Foundation Trust, Dudley, UK; 10 Cancer Clinical Trials Unit, University of Birmingham, Birmingham, UK; 11 National Clinical Lead for Organ Donation, NHS Blood and Transplant, Bristol, UK

**Keywords:** Cost-effectiveness of adrenaline, Cardiac arrest, Organ donation, Economics

## Abstract

**Background:**

The ‘Prehospital Assessment of the Role of Adrenaline: Measuring the Effectiveness of Drug Administration In Cardiac Arrest’ (PARAMEDIC2) trial showed that adrenaline improves overall survival, but not neurological outcomes. We sought to determine the within-trial and lifetime health and social care costs and benefits associated with adrenaline, including secondary benefits from organ donation.

**Methods:**

We estimated the costs, benefits (quality-adjusted life years (QALYs)) and incremental cost-effectiveness ratios (ICERs) associated with adrenaline during the 6-month trial follow-up. Model-based analyses explored how results altered when the time horizon was extended beyond 6 months and the scope extended to include recipients of donated organs.

**Results:**

The within-trial (6 months) and lifetime horizon economic evaluations focussed on the trial population produced ICERs of £1,693,003 (€1,946,953) and £81,070 (€93,231) per QALY gained in 2017 prices, respectively, reflecting significantly higher mean costs and only marginally higher mean QALYs in the adrenaline group. The probability that adrenaline is cost-effective was less than 1% across a range of cost-effectiveness thresholds. Combined direct economic effects over the lifetimes of survivors and indirect economic effects in organ recipients produced an ICER of £16,086 (€18,499) per QALY gained for adrenaline with the probability that adrenaline is cost-effective increasing to 90% at a £30,000 (€34,500) per QALY cost-effectiveness threshold.

**Conclusions:**

Adrenaline was not cost-effective when only directly related costs and consequences are considered. However, incorporating the indirect economic effects associated with transplanted organs substantially alters cost-effectiveness, suggesting decision-makers should consider the complexity of direct and indirect economic impacts of adrenaline.

**Trial registration:**

ISRCTN73485024. Registered on 13 March 2014.

## Introduction

Although survival rates following adult out-of-hospital cardiac arrest have increased in recent years, they generally remain below 10% [1, 2]. Survivors are at increased risk of cognitive and functional impairment and often take significant time to attain an acceptable quality of life or to return to work [3].

In some countries, when a patient dies after cardiac arrest, families can be asked to agree to the patient’s organs being donated for the benefit of others. A meta-analysis of 26 studies reported that, on average, 5.8% (95% CI 2.1–10.9%) of patients admitted to intensive care donate their organs for transplantation after brain stem death [4]. Observational studies suggest that cardiac arrest before organ donation does not adversely affect long-term graft function [5] and that such transplant programmes are cost-effective [6, 7].

The PARAMEDIC2 (Prehospital Assessment of the Role of Adrenaline: Measuring the Effectiveness of Drug Administration In Cardiac Arrest) trial showed that adrenaline improves overall survival, but not long term neurological outcome [8, 9]. In this paper, we explore the costs and benefits of adrenaline within the PARAMEDIC2 trial across within-trial and lifetime horizons and model the contribution of organ donation after a cardiac arrest on cost-effectiveness.

## Methods

### Trial background

PARAMEDIC2 (ISRCTN73485024) was a pragmatic, individually randomised, double-blind, controlled trial of 8014 adult patients with out-of-hospital cardiac arrest in the UK [9]. Patients, treated by five National Health Service (NHS) ambulance services between December 2014 and October 2017, were randomised to either parenteral adrenaline or saline placebo, along with standard care. The primary clinical outcome was the rate of survival at 30 days. Survival, neurological outcomes as indicated by the modified Rankin Scale (mRS) (ranging from 0 [no symptoms] to 6 [death]) [10] and economic outcomes were followed up from randomisation to 6 months. The South Central–Oxford C Research Ethics Committee (REC: 14/SC/0157) and the Medicines and Healthcare Products Regulatory Authority (EudraCT: 2014-000792-11) approved the study protocol. The trial was funded by the UK National Institute for Health Research Health Technology Assessment Programme (12/127/126). Further details of the trial are reported elsewhere [9].

### Overview of economic evaluation

A cost-utility analysis was conducted, with results expressed in terms of incremental cost per quality-adjusted life year (QALY) gained through the use of adrenaline. The baseline economic evaluation was conducted from the perspective of the UK NHS and personal social services (PSS) [11], with a time horizon of 6 months post-randomisation. A decision-analytic model was used to extrapolate outcomes beyond trial follow-up and assess the cost-effectiveness of adrenaline over a lifetime horizon. All costs were expressed in British pounds sterling and valued at 2017 prices (with commensurate values in Euros estimated using purchasing power parities). Costs and QALYs accrued beyond the first year of follow-up were discounted to present values at 3.5% per annum in accordance with recommendations from the National Institute for Health and Care Excellence (NICE) [11]. The economic evaluation adhered to an approved pre-specified analytical plan which is available in the supplementary material (Additional file 1).

### Measurement and valuation of resource use

Resource inputs associated with the pre-hospital emergency response, until the point of hospital admission or death, were extracted from the trial case report forms completed by research paramedics. These data included the number of emergency response staff/ambulance crew and vehicles in attendance, duration of emergency response, and cumulative doses of adrenaline administered. Unit costs for these resource inputs were drawn from national tariffs (Additional file 2).

For patients surviving to hospital admission, the economic costs associated with hospital care were extracted through data linkage with two national population-wide databases: Hospital Episode Statistics (HES) for England and the Patient Episode Database for Wales (PEDW). This provided patient-level profiles of resource use associated with hospital episodes for study patients over the trial time horizon, including emergency department attendances, critical care and other inpatient admissions, covering procedures undertaken including percutaneous coronary intervention, by-pass surgery, implantable cardioverter defibrillators (ICDs), cardiac pacemakers and lengths of stay, day case admissions and outpatient attendances. HealthCare Resource Group (HRG) codes were derived using the NHS HRG4 2016–17 Reference Cost Grouper software version [12]. The Department of Health and Social Care’s *Reference Costs 2016–17* schedule was used to assign costs to derived HRG codes [12]. For patients without linkage to routine hospital records, hospital resource inputs were extracted from data contained within the trial case report forms and valued using national tariffs.

Surviving patients or their proxies were asked to complete questionnaires at 3 and 6 months post-randomisation, reporting hospital admissions and use of hospital outpatient services following initial discharge, by type and volume, and use of community health and social services, medications and aids and equipment. Further questions captured wider societal attributable resource use, with data collected on time off work, over-the-counter medications, aids and adaptations purchased privately and any additional costs borne by study patients or informal carers. Unit costs for these resource inputs were drawn from national tariffs (Additional file 2).

### Calculation of health utilities and QALYs

Health-related quality of life was assessed using the EuroQol EQ-5D-5L [13], completed at 3 and 6 months post-cardiac arrest. Responses to the EQ-5D-5L descriptive system were converted into health utility values, based on the UK EQ-5D-3L tariff using the van Hout interim cross-walk algorithm [14], in line with current recommendations [15]. mRS scores collected at hospital discharge were also converted into health utility values, based on the UK EQ-5D-3L tariff, using a published mapping algorithm [16]. A QALY profile was generated for each trial participant using the area-under-the-curve method, assuming a baseline health utility value of zero [17] and linear interpolation between utility measurements at hospital discharge and at 3 and 6 months assessment points, accounting for survival [18]. We used 3- or 6-month mRS-derived health utilities in place of EQ-5D-5L-derived values if the latter were missing.

### Analytical methods

#### Analyses of clinical outcomes, resource use and costs

Survival outcomes were analysed through the use of fixed-effect regression models with adjustment for age, sex, interval between the emergency call and the ambulance arrival at the scene, interval between the ambulance arrival and the administration of the study drug, cause of cardiac arrest, initial cardiac rhythm, whether the cardiac arrest was witnessed and whether CPR was performed by a bystander [9]. Between treatment-group differences in mean resource use and mean costs were estimated using two-sample Student’s *t* tests. Estimates of 95% confidence intervals (CIs) surrounding between-group differences in mean costs were obtained using nonparametric bootstrapping with replacement, based on 1000 replications [19].

#### Handling missing data

Multiple imputations using chained equations with predictive mean matching [20] was used to predict values for missing observations, assuming data were missing at random. Missing costs and health utility values were imputed at the level of resource category, health-related quality of life measure and assessment point, stratified by survival status at hospital discharge and treatment allocation in accordance with current recommendations [21]. Twenty imputed datasets were generated and used to inform the base-case and subsequent sensitivity and subgroup analyses. Parameter estimates were pooled across the 20 imputed datasets using Rubin’s rule to account for between and within-imputation components of variance terms associated with parameter estimates.

#### Within-trial economic evaluation

Bivariate linear regressions that take into account the correlation between each patient’s costs and effects were used to model total costs and total QALYs over the 6-month follow-up period. By specifying the treatment group as an indicator within each equation, the incremental costs and QALYs attributable to adrenaline were estimated, while controlling for baseline covariates (age, sex, time to first dose administration, cause of cardiac arrest, whether the cardiac arrest was witnessed, whether a bystander performed CPR and initial cardiac rhythm). Cost-effectiveness was expressed in terms of an incremental cost-effectiveness ratio (ICER), defined as the incremental adjusted cost of adrenaline divided by the incremental adjusted QALYs of adrenaline. The ICER was then compared with a range of cost-effectiveness threshold values for an additional QALY. ICERs falling below or in the region of £20,000 (€23,000) to £30,000 (€34,500) per QALY gained would, in general, be considered as representing a cost-effective use of UK NHS resources [11, 22]. Confidence intervals surrounding mean ICER values were not calculated as they are problematic to interpret if the ICER denominator is estimated close to zero and the interval extends to cover negative values [23]. This is because a negative ICER might equally imply lower costs and more effective treatment or greater costs and less effective treatment compared with placebo. There is no way of differentiating between these two qualitatively different scenarios from a confidence interval alone. Thus, we followed standard practice in economic evaluations to present mean ICER values without accompanying confidence intervals or standard errors. Instead, uncertainty surrounding mean cost-effectiveness estimates were characterised through a Monte Carlo method involving simulating 1000 replicates of the ICER from a joint distribution of incremental costs and incremental QALYs [24]. By calculating net monetary benefits for each of these simulated ICER values at alternative cost-effectiveness thresholds varying from £0 to £100,000 (€115,000) per QALY gained, the probability of cost-effectiveness of adrenaline (defined as the proportion of positive net monetary benefits at a given threshold level) was calculated and plotted as a cost-effectiveness acceptability curve [25].

#### Sensitivity analyses

A series of sensitivity analyses, summarised in Additional file 3, assessed the impact of varying features of the within-trial economic evaluation on cost-effectiveness. These included extending the perspective of analysis to cover broader societal resource use and costs and extending the time horizon to 12 months after the cardiac arrest event. Further economic modelling (described in the sections that follow) explored how results altered when the time horizon was extended to cover the lifetimes of cardiac arrest survivors and the scope extended to include recipients of donated organs.

#### Sub-group analyses

A series of pre-specified subgroup analyses, summarised in Additional file 4, explored whether the cost-effectiveness estimates based on the within-trial data altered by age, gender, time to first dose administration, cause of cardiac arrest, whether the cardiac arrest was witnessed, whether bystander performed CPR or initial cardiac rhythm.

#### Extrapolations of cost-effectiveness

**Fig. 1:**
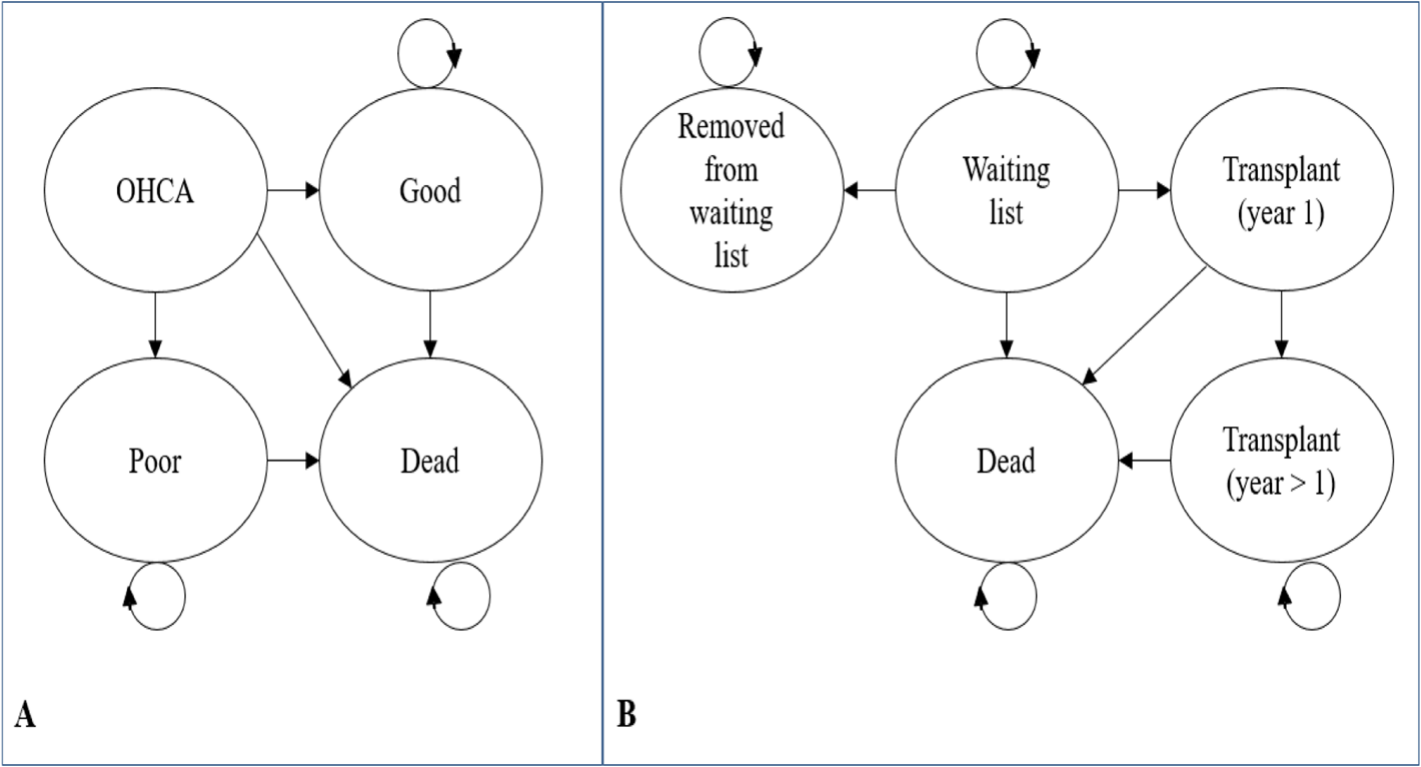
Model diagrams. Plot **a**: Model to extrapolate cost-effectiveness beyond PARAMEDIC2 trial follow-up. OHCA represents health state for out-of-hospital cardiac arrest patients. Good represents survival with good neurological function. Poor represents survival with poor neurological function. Plot **b**: Organ donor model adapted from Fisher et al. [26]

Controlled organ donation after neurological or cardiac death is available to families for patients who sustain un-survivable severe brain injury. Decision-analytic modelling was used to estimate the incremental cost-effectiveness of adrenaline beyond the parameters of the PARAMEDIC2 trial to include the costs and benefits of organ donation. A Markov state transition model was developed to extrapolate lifetime costs and benefits for out-of-hospital cardiac arrest survivors (Fig. 1, plot a). We separately adapted a previous economic model of adult lung transplantation [26] to simulate the impact on the cost-effectiveness of adrenaline resulting from changes in organ donation from deceased PARAMEDIC2 donors who had experienced out-of-hospital cardiac arrest. The model (Fig. 1, plot b) simulated pre and post-transplantation survival for individuals on the active national transplant waiting list. Epidemiological data for the model were informed by a separate linkage of the PARAMEDIC2 patients to the UK National Transplant Registry. Further details of the model structures, data inputs, analytical methods and description of methods for integrating the direct and indirect effects of adrenaline generated by the two models are provided in Additional file 5.

## Results

A total of 8014 patients were randomised to either parenteral adrenaline (4015 patients) or saline placebo (3999 patients). In the adrenaline group, 130 patients (3.2%) were alive at 30 days, compared with 94 patients (2.4%) in the placebo group (unadjusted odds of survival, 1.39; 95% confidence interval [CI], 1.06 to 1.82; *P* = 0.02) [9]. Severe neurologic impairment at hospital discharge (mRS score of 4 or 5) was more common amongst survivors in the adrenaline group than in the placebo group (39 of 126 patients [31.0%] vs. 16 of 90 patients [17.8%]) [9].

**Fig. 2:**
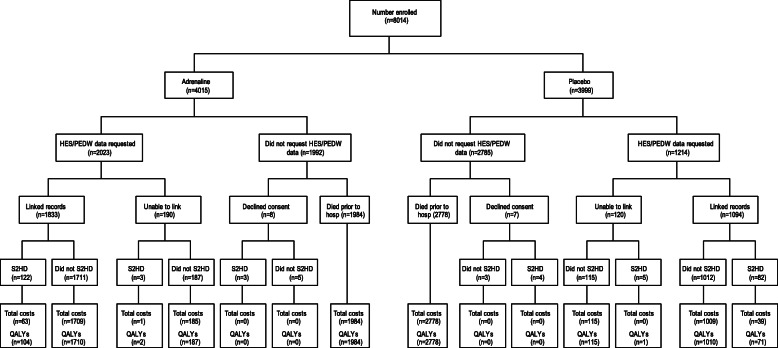
Flow chart of data sources used to inform resource use and costs. S2HD indicates survivors to hospital discharge, HES/PEDW indicates Hospital Episode Statistics/Patient Episode Database for Wales

Figure 2 summarises the flow and completeness of data sources used to inform the economic parameters of the trial. Overall, between 1 and 2% of costs (at the component level) and approximately 1% of QALY data were missing (and subsequently imputed) for the primary analysis.

Resource use reported by the trial participants and or their proxies by treatment arm is summarised in Additional file 6. More patients were admitted to intensive care in the adrenaline arm (2016 vs 1209, *P* < 0.001), creating a larger group for assessment for donation after neurological or cardiac death. Linkage of the PARAMEDIC2 patients to the UK National Transplant Registry revealed that there were 99 recipients of organs donated by the adrenaline group compared with 67 recipients of organs donated by the placebo group (from 40 donors in the adrenaline group vs 24 donors in the placebo group). Breakdown by organ type is provided in Table 1.

**Table 1:** PARAMEDIC2 organ donor and recipient information

		Placebo	Adrenaline	Total	*P* value
Number of recipients		67	99	166	
Number of organs donated		74	115	189	
Gender of donor	Males	14 (58.3%)	24 (60.0%)	38 (59.4%)	0.895
	Females	10 (41.7%)	16 (40.0%)	26 (40.6%)	
Gender of recipient	Males	37 (55.2%)	61 (61.6%)	98 (59.0%)	0.411
	Females	30 (44.8%)	38 (38.4%)	68 (41.0%)	
Age of the recipient	< 18	4 (6.0%)	5 (5.1%)	9 (5.4%)	0.748
	18–30	6 (9.0%)	6 (6.1%)	12 (7.2%)	
	31–50	25 (37.3%)	30 (30.3%)	55 (33.1%)	
	51–60	15 (22.4%)	32 (32.3%)	47 (28.3%)	
	61–70	14 (20.9%)	22 (22.2%)	36 (21.7%)	
	> 70	3 (4.5%)	4 (4.0%)	7 (4.2%)	
Average number of organs (per donor)	*N*^1^	24	40	64	
	Mean	3.1	2.9	3.0	0.584
	Median	3	3	3	
	Std	1.6	1.2	1.4	
	Min-max	1–7	1–6	1–7	
	Missing	0	0	0	
Type of organs (*n*)	Kidney only	39 (58.2%)	54 (54.5%)	93 (56.0%)	0.457
	En-bloc kidney	0 (0.0%)	0 (0.0%)	0 (0.0%)	
	Double kidney	0 (0.0%)	5 (5.1%)	5 (3.0%)	
	Heart only	3 (4.5%)	4 (4.0%)	7 (4.2%)	
	Liver only	16 (23.9%)	24 (24.2%)	40 (24.1%)	
	Pancreas only	2 (3.0%)	0 (0.0%)	2 (1.2%)	
	Single lung	0 (0.0%)	1 (1.0%)	1 (0.6%)	
	Double lung	3 (4.5%)	3 (3.0%)	6 (3.6%)	
	Liver and kidney	1 (1.5%)	1 (1.0%)	2 (1.2%)	
	Kidney and pancreas	3 (4.5%)	7 (7.1%)	10 (6.0%)	

^1^*N* refers to the number of donors in adrenaline and placebo groups

Amongst all participants, mean total NHS and PSS costs over the 6-month trial time horizon was £3789 (€4357) in the adrenaline group vs. £2698 (€3103) in the placebo group [unadjusted mean cost difference £1091 (€1255) (bootstrap 95% CI £807 (€928) to £1398 (€1608)); *P* < 0.001] (Table 2). There was also a significant difference in mean total societal costs: £3829 (€4403) in the adrenaline group vs. £2687 (€3090) in the placebo group [unadjusted mean cost difference £1143 (€1314) (bootstrap 95% CI £861 (€990) to £1451 (€1669)); *P* < 0.001]. Amongst those who survived to hospital discharge, there were no significant differences in mean total NHS and PSS costs. However, amongst survivors to hospital discharge, mean utility values were significantly lower at hospital discharge in the adrenaline group [0.48 vs 0.60, unadjusted mean difference − 0.118 (95% CI − 0.196 to − 0.032); *P* = 0.002].

**Table 2:** Total costs in 2017 prices by trial arm

Assessment period	Category	Adrenaline (***n*** = 4015)		Placebo (***n*** = 3999)		Adrenaline versus placebo	
		*N*	Mean (SE), £	*N*	Mean (SE), £	Mean cost difference (bootstrap 95% CI), £	*P* value
0–6 months	Hospitalisation costs (NHS and PSS)	4006	2669 (179)	3988	1460 (129)	1209 (804, 1660)	< 0.001
	Non-hospitalisation costs (NHS and PSS)	3934	1785 (21)	3926	1678 (13)	107 (61, 155)	< 0.001
	Non-NHS and PSS costs	3956	51 (15)	3952	15 (6)	36 (8, 69)	0.01
	Total NHS and PSS costs	3934	3789 (121)	3926	2698 (94)	1091 (807, 1398)	< 0.001
	Total societal costs	3933	3829 (124)	3925	2687 (92)	1143 (861, 1451)	< 0.001

Data are for all patients and based on combined HES© and CRF data. Copyright© (2019), the Health and Social Care Information Centre. Re-used with the permission of the Health and Social Care Information Centre [and/or [name of licensor]]. All rights reserved

*CI* confidence interval, *N* number of participants with complete data (zero utilisation and costs assigned to deaths), *NHS* National Health Service, *PSS* Personal Social Services, *SE* standard error

**Fig. 3:**
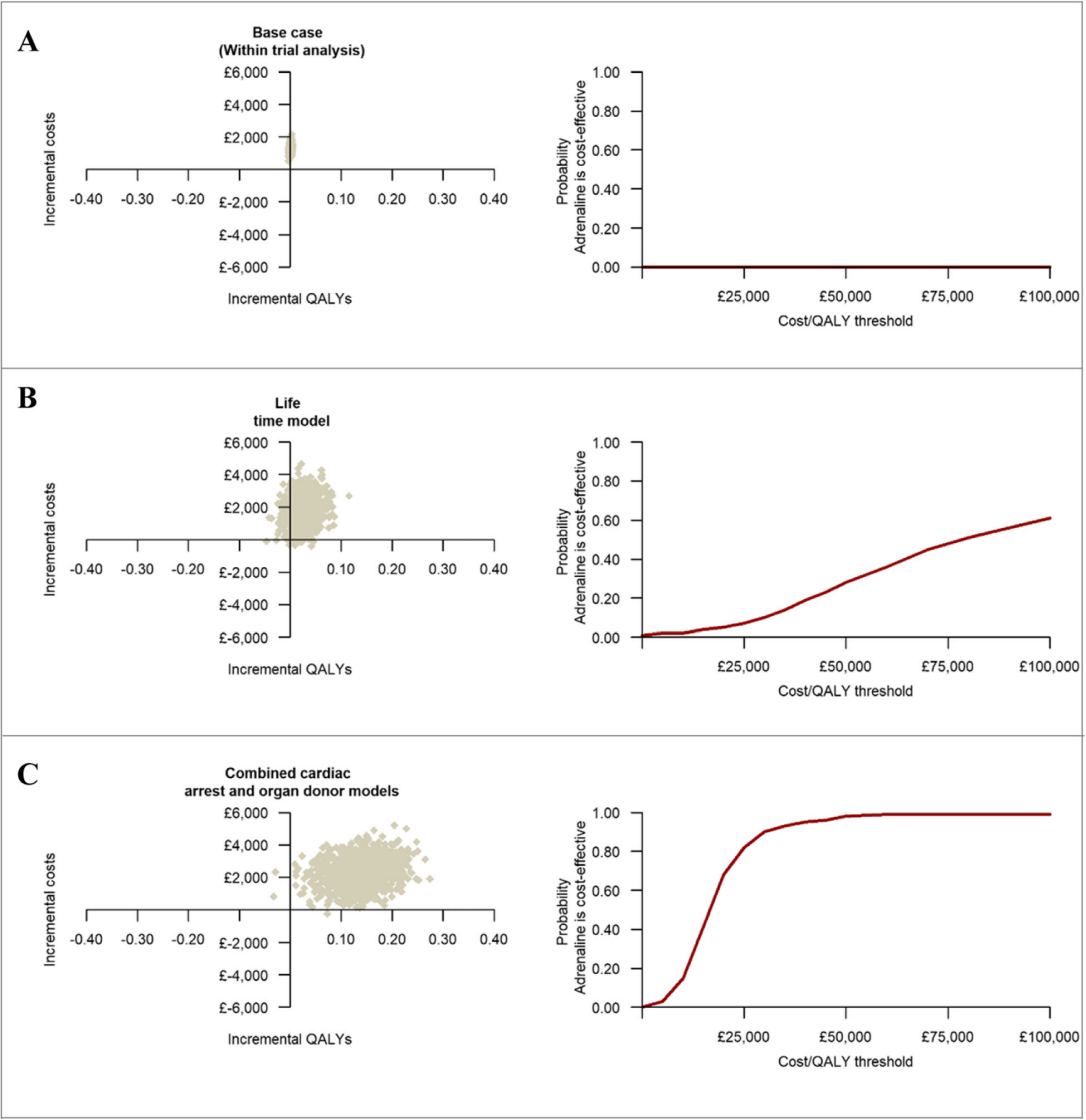
The three graphs on the left-hand side represent the cost-effectiveness plane for the within-trial, lifetime and the combined cardiac arrest and organ donor analyses. The three graphs on the right-hand side represent cost-effectiveness acceptability curves and give the probability that adrenaline is cost-effective compared with placebo at a specified cost-effectiveness threshold. Each cost-effectiveness plane is divided into four quadrants (North-West, North-East, South-West and South-East) by the intersection of the horizontal and vertical axis. South-East quadrant implies adrenaline is less costly and more effective than placebo, North-West quadrant implies adrenaline is less effective and more costly, the North-East quadrant implies adrenaline is more effective but also more costly and the South-West quadrant implies adrenaline is less effective but also less costly

The within-trial economic evaluation results are summarised in Table 3. The base case analysis, over a 6-month time horizon, produced an ICER of £1,693,003 (€1,946,953) per QALY gained, reflecting significantly higher mean costs and only marginally higher mean QALYs in the adrenaline group. Extrapolating the within-trial analysis produced ICERs of £644,308 (€740,954) per QALY gained over 1 year and £81,070 (€93,231) per QALY gained over the lifetimes of survivors. The probability that adrenaline is cost-effective remained below 1% across a range of cost-effectiveness thresholds (plots a and b of Fig. 3), while mean net monetary benefits were negative at all thresholds. The cost-effectiveness estimates remained robust to a range of sensitivity and subgroup analyses (Additional file 7).

**Table 3:** Base-case within-trial cost-effectiveness results

Analytical model*	Adrenaline		Placebo		Cost-effectiveness			Incremental net monetary benefit at cost-effectiveness of			Probability adrenaline is cost-effective at		
	Mean costs (SE)	Mean QALYs (SE)	Mean costs (SE)	Mean QALYs (SE)	Incremental costs (95%CI)	Incremental QALYs (95%CI)	ICER (£ per QALY)	£15,000 per QALY (95% CI)	£20,000 per QALY (95% CI)	£30,000 per QALY (95% CI)	£15,000 per QALY	£20,000 per QALY	£30,000 per QALY
Within-trial base-case*, 6 months post-cardiac arrest event	£3591 (£540)	0.0025 (0.0025)	£2285 (£544)	0.0017 (0.0025)	£1306 (£837, £1774)	0.0008 (− 0.0014, 0.003)	£1,693,003	− £1294 (− £1753, − £835)	− £1290 (− £1746, − £834)	− £1282 (− £1733, − £831)	0	0	0
Extrapolation to 12 months post-cardiac arrest event	£3741 (£536)	0.006 (0.0049)	£2330 (£541)	0.0038 (0.005)	£1411 (£946, £1876)	0.0022 (− 0.0021, 0.0065)	£644,308	− 1378 (− 1826, − 931)	− £1367 (− £1811, − £924)	− £1346 (− £1784, − £907)	0	0	0
Extrapolation to lifetime horizon (decision analytic model)	£5308 (£797)	0.111 (0.037)	£3534 (£736)	0.089 (0.031)	£1775 (£250, £3394)	0.022 (− 0.011, 0.063)	£81,070	− 1445 (− £2996, £39)	− £1335 (− £2945, £179)	− £1155 (− £2832, £521)	0	0	0.098

*CI* confidence interval, *ICER* incremental cost-effectiveness ratio, *INMB* incremental net monetary benefit, *MI* multiple imputation, *pCE* probability cost-effective, *QALYs* quality-adjusted life years, *SE* standard error

*Adjusted multiple imputation analyses (assumed missing data missing at random) and accounting for patient age, sex, interval between emergency call and ambulance arrival at scene, interval between ambulance arrival at scene and administration of the trial agent, initial cardiac rhythm, cause of cardiac arrest, whether the cardiac arrest was witnessed and whether a bystander performed CPR

The decision-analytic modelling that estimated combined direct economic effects over the lifetimes of survivors and indirect economic effects in organ recipients revealed that routine use of adrenaline at a UK-wide level in this patient group would result in incremental costs of £49,759,481 (€57,223,403) and 3093 additional QALYs per annum (Table 4). The mean ICER at a patient-level was estimated at £16,086 (€18,499) per QALY gained for adrenaline. The probability that adrenaline is cost-effective compared with placebo was 39% at a £15,000 (€17,250) per QALY cost-effectiveness threshold, increasing to 90% at a £30,000 (€34,500) threshold (Table 4 and plot c of Fig. 3). Sensitivity analyses revealed that these cost-effectiveness estimates remained robust to a range of modelling assumptions and data inputs with the exception of the estimated impact of the number of organs donated for transplantation on the cost-effectiveness of adrenaline (Additional file 5).

**Table 4:** Base-case results combining direct and indirect effects of adrenaline use in cardiac arrest

Analysis	Adrenaline		Placebo		Cost-effectiveness			Probability adrenaline is cost-effective at		
	Mean costs	Mean QALYs	Mean costs	Mean QALYs	Incremental costs	Incremental QALYs	ICER	£15,000 per QALY	£20,000 per QALY	£30,000 per QALY
Trial-based analysis (*n* = 21,757)^1^	£78,130,649	54	£48,662,440	37	£29,468,209	17	£1,693,003	0.00	0.00	0.00
Trial-based + extrapolation (*n* = 21,757) ^1^	£115,497,429	2414	£76,885,801	1938	£38,611,628	476	£81,070	0.04	0.05	0.10
Trial-based + extrapolation + lung (*n* = 81)	£120,803,455	2642	£82,061,552	2138	£38,741,903	504	£76,859	0.03	0.05	0.10
Trial-based + extrapolation + liver (*n* = 309)	£163,048,181	5452	£116,865,746	4187	£46,182,436	1264	£36,533	0.06	0.13	0.35
Trial-based + extrapolation + kidney (*n* = 731)	£262,782,220	9676	£220,723,823	7398	£42,058,397	2278	£18,466	0.34	0.57	0.82
Trial-based + extrapolation + lung + liver + kidney	£315,638,999	12,942	£265,879,518	9849	£49,759,481	3093	£16,086	0.41	0.68	0.90

Results are presented at the UK population level and are for the combined analysis population of patients who stand to benefit directly and indirectly from adrenaline use in cardiac arrest

*ICER* incremental cost-effectiveness ratio

^1^*n* refers to the eligible UK-wide out-of-hospital cardiac arrest patient population per year predicted from the modelling; the transplant estimates refers to predicted numbers joining waiting list each year

## Discussion

This paper presents the first economic evaluation of adrenaline in adults with out-of-hospital cardiac arrest. The study revealed that when the assessment is restricted to the first 6 months that follow out-of-hospital cardiac arrest, adrenaline is associated with significantly higher mean costs, largely driven by the additional costs associated with hospital admissions, and only marginally higher mean QALYs. The base case analysis generated a mean ICER of £1,693,003 (€1,946,953) per QALY gained at 6 months post-randomisation, well in excess of accepted cost-effectiveness thresholds [11]. Consequently, the probability that adrenaline is cost-effective approximated to zero in the base-case analysis. Moreover, this conclusion remained robust after extensive sensitivity analyses that accounted for different sources of uncertainty. Notably, however, separate decision-analytic modelling that extrapolated cost-effectiveness over the lifetimes of survivors and incorporated additional costs and health consequences for organ recipients significantly altered the cost-effectiveness calculus. The probability that adrenaline is cost-effective increased to 90% at a £30,000 (€34,500) per QALY cost-effectiveness threshold, arguably within the bounds of acceptance by health technology agencies tasked with reimbursement decisions [11]. The cost-effectiveness conclusions were particularly sensitive to the inclusion of recipients of donated organs into economic modelling. The economic value of the additional survival and QALY benefits to organ recipients associated with adrenaline clearly outweigh the additional costs that result from organ transplantation. Similar findings have been reported for other interventions. In a study evaluating the costs and benefits of traumatic cardiopulmonary arrest resuscitation [27], the authors concluded that the financial burden associated with the procedure could be offset by expanding the benefits to include organ donation.

Previous economic evaluations of adrenaline were conducted in clinical contexts outside the management of out-of-hospital cardiac arrest [28–30], and therefore, a comparative assessment of cost-effectiveness evidence is not possible. Similarly, other pharmacological interventions for cardiac arrest, such as anti-arrythmics, have yet to be evaluated from an economic perspective. Our data should, therefore, be of relevance to clinical decision-makers and service planners tasked with the care and management of a condition that causes significant death and disability. Moreover, our economic evaluation is the first, to our knowledge, that has modelled the indirect economic benefits of organ donation arising from an intervention that increases the likelihood of admission to intensive care following an acute out-of-hospital clinical event. It may, therefore, alter methodological thinking on the incorporation of benefits other than directly to the patient into economic evaluations of interventions that affect the likelihood for organ donation. Such a methodological shift will require ethical consideration of how to balance benefits to others with benefits or harms to patients receiving the intervention.

Readers should consider caveats when interpreting the study results. First, our findings may not be applicable outside of the UK as the models of prehospital care, and healthcare costs may differ in other countries. Second, our adapted model of adult lung transplantation excluded the effects of adrenaline on pancreas and heart transplants. A paucity of epidemiological and economic evidence meant that we were unable to parameterise extensions to our decision-analytic model that considered effects beyond lung, liver and kidney transplants. Nevertheless, the number of pancreases and hearts transplanted to recipients of organs donated by the PARAMEDIC2 patients was small (< 10) and therefore unlikely to alter the balance of overall conclusions. Third, as with any health economic evaluation, there are likely to be potentially relevant effects that are not captured by the tools available for measurement and valuation. Specifically, we did not value other potential consequences of adrenaline, for example, the potential benefits to family members arising from the increased likelihood of being able to say goodbye and be present at the time of death [31]. Finally, in the modelling that extended our assessments of cost-effectiveness of adrenaline over the lifetimes of cardiac arrest survivors and incorporated the indirect economic effects of organ transplantation, a number of assumptions were required to simplify the parameterisation and address data limitations. Details of these assumptions are described in additional file 5. These included assuming that functional health state does not change over extended periods of survival and that resource use captured in the 3–6 month post-randomisation period can be extrapolated to cover the 6–12 month post-randomisation period. The organ donor modelling required a set of steady or equilibrium-state assumptions (number of organs available each year equals the number of transplantations with no wastage; the probability of death or removal on the waiting list are independent of organ supply) that captured the impact of intervention on the probability of transplantation. Nevertheless, our approach was consistent with current methodological guidance for decision-analytic modelling [32]. Furthermore, extensive sensitivity analyses were conducted that found that the conclusions from the base-case analyses were robust to doubling and halving input parameter values and alternative specifications of modelling assumptions.

## Conclusion

In conclusion, adrenaline use in adults with out-of-hospital cardiac arrest does not represent a cost-effective use of resources when only directly related costs and consequences are considered. However, incorporating the indirect economic effects associated with transplanted organs substantially alters the cost-effectiveness calculus, suggesting decision-makers should consider the complexity of direct and indirect economic impacts of adrenaline.

## Supplementary information

**Additional file 1.** PARAMEDIC2 HEALTH ECONOMICS ANALYSIS PLAN. Details of the PARAMEDIC2 health economics analysis plan.

**Additional file 2.** Unit costs for NHS, non-NHS and personal and social service resource inputs (£ sterling, 2017 prices). Unit costs data used to inform the within trial economic evaluation.

**Additional file 3.** Pre-specified and post-hoc sensitivity analyses exploring varying features of the within-trial economic evaluation on the cost-effectiveness results. List of sensitivity analyses.

**Additional file 4.** Subgroup analyses conducted that explored potential heterogeneity in the within-trial incremental cost-effectiveness of adrenaline.

**Additional file 5.** Economic modelling to extrapolate cost-effectiveness beyond trial follow-up and incorporate the indirect effects of organ donation. List of subgroup analyses.

**Additional file 6.** Resources use for NHS and personal and social services by trial arm in the 6 month post-randomisation period; all patients. Resource use results table, within-trial economic evaluation.

**Additional file 7.** Cost-effectiveness (£, 2017 prices) of adrenaline based on within-trial economic evaluation; sensitivity analyses and subgroup analyses results. Additional cost-effectiveness results.

## Data Availability

Not applicable

## Abbreviations

DBD: Donation after brain death
DCD: Donation after circulatory death
EVLP: Ex vivo lung perfusion
EVMP: Ex vivo machine perfusion
HES: Hospital Episode Statistics
HRG: HealthCare Resource Group
ICD: Implantable cardioverter defibrillator
ICER: Incremental cost-effectiveness ratio
NHS: National Health Service
NHS/PSS: NHS and Personal Social Services
NICE: National Institute for Care Excellence
PEDW: Patient Episode Database for Wales
QALY: Quality-adjusted life year
UK: United Kingdom
UKBT: United Kingdom Blood and Transplant
