# Supplementary material for: Cost-effectiveness of adrenaline for out-of-hospital cardiac arrest
Source: Crit Care. 2020 Sep 27;24:579. doi: 10.1186/s13054-020-03271-0 (PMC7520962; doi:10.1186/s13054-020-03271-0)
Supplement: Supplementary file 1 — Additional file 1. PARAMEDIC2 HEALTH ECONOMICS ANALYSIS PLAN. Details of the PARAMEDIC2 health economics analysis plan. [file 13054_2020_3271_MOESM1_ESM.zip › 2020-06-27-Additional file 1.docx]

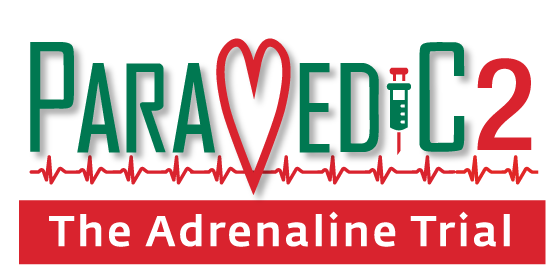


**ADDITIONAL FILE 1: PARAMEDIC2 HEALTH ECONOMICS ANALYSIS PLAN**

**Prehospital Assessment of the Role of Adrenaline: Measuring the Effectiveness of Drug administration In Cardiac arrest**

| EudraCT Number: | 2014-000792-11 |
| --- | --- |
| ISRCTN: | ISRCTN73485024 |
| Funding Body: | NIHR Health Technology Assessment Programme |
| Ethics Approval: | Oxford C REC: 14/SC/0157 Date: 21^st^ May 2014 |
|  |  |
| Version number: | 1.0 |
| Date: | 13^th^ April 2018 |
| Stage: | FINAL |

Chief Investigator signature: ______________________

**SAP Amendments:**

| **Amendment Number:** | **Date of Amendment:** | **Date of Approval:** |
| --- | --- | --- |
|  |  |  |
|  |  |  |
|  |  |  |
|  |  |  |
|  |  |  |
|  |  |  |


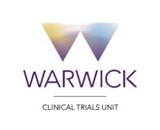


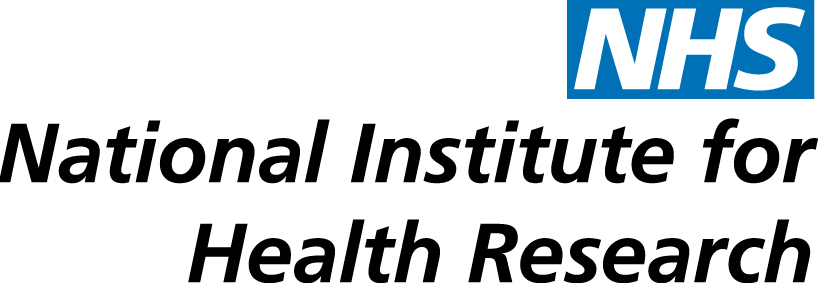


**CONTENTS**

[Objective 2](#_Toc511379710)

[Introduction/background epidemiology 2](#_Toc511379711)

[General principles for economic evaluation 3](#_Toc511379712)

[Resource use and costs 3](#_Toc511379713)

[Outcomes 4](#_Toc511379714)

[Data quality and cleaning 6](#_Toc511379715)

[Missing data 6](#_Toc511379716)

[Cost-effectiveness analysis 7](#_Toc511379717)

[Result tables 8](#_Toc511379718)

[Reference 32](#_Toc511379719)

# Objective

The aim of the within-trial economic evaluation is to estimate the cost-effectiveness of adrenaline use compared with placebo in out-of-hospital cardiac arrest. The evaluation is being conducted alongside the Pre-hospital Assessment of the Role of Adrenaline: Measuring the Effectiveness of Drug administration In Cardiac arrest (PARAMEDIC-2) trial. PARAMEDIC-2 is a pragmatic, two-armed individually randomised, double blind, placebo controlled trial with a parallel economic evaluation. The purpose of the health economics analysis plan is to outline an explicit framework of methods that will be used to analyse the health economic data in a robust manner.

# Introduction/background epidemiology

Adrenaline has been an integral component of advanced life support from the birth of modern cardiopulmonary resuscitation in the early 1960s. In guidelines written originally in 1961, Peter Safar recommended the use of very large doses of adrenaline: 10 mg intravenously or 0.5 mg intra-cardiac ([1](#_ENREF_1)) and adrenaline has continued to be recommended ever since. The International Liaison Committee on Resuscitation (ILCOR) synthesised the available evidence for adrenaline in 2010 ([2](#_ENREF_2)) and re-assessed the evidence in October 2015 ([3](#_ENREF_3)) noting that whilst it may improve the return of spontaneous circulation (ROSC) and short-term survival, there is insufficient evidence to know if adrenaline had beneficial or harmful effects on survival to discharge from hospital and on neurological outcomes. ILCOR has called for placebo-controlled trials to evaluate the use of any vasopressor in adult and paediatric cardiac arrest.

The use of adrenaline in cardiac arrest increases the chances that the heart is restarted [ROSC] but there remains doubt as to whether this is translated into improved or worse long term survival and neurological outcomes. The PARAMEDIC-2 trial seeks to establish whether the use of intravenous adrenaline, administered in accordance with current cardiac arrest guidelines is clinically-effective and cost-effective treatment for out-of-hospital cardiac arrest.

# General principles for economic evaluation

The within-trial economic analysis will be conducted under the intention to treat (ITT) principle which requires study participants to be analysed according to their treatment assignment irrespective of actual treatment received ([4](#_ENREF_4)). The perspective of the base case analysis will be that of the UK health and personal social care services (NHS/PSS) as recommended by National Institute for Health and Care Excellence (NICE) reference case for appraising health technologies ([5](#_ENREF_5)). Secondary analyses will consider costs from a wider societal perspective ([6](#_ENREF_6)). A 6 month time horizon will be adopted to match the trial follow-up period covering the 6 months period following out-of-hospital cardiac arrest. As a result, no discounting will be applied to costs and outcomes in the within-trial analysis. However, if longer term decision modelling were to be undertaken, then costs and outcomes will be discounted at 3.5% beyond the first year post randomisation in accordance with the NICE reference case ([5](#_ENREF_5)). The findings of this economic evaluation will be reported in accordance with the Consolidated Health Economic Evaluation Reporting Standards (CHEERS) statement for the reporting of health economic evaluations ([7](#_ENREF_7)).

# Resource use and costs

Data will be collected on the health and social service use and costs for each trial participant during the period between randomisation and six months post-randomisation. Resource utilisation data will be collected through four principal means: (i) use of trial interventions, concurrent treatments, mode and distance of initial transportation and subsequent transfers, will be estimated using the computerised data collection systems developed for the PARAMEDIC trial; (ii) detailed information on ITU resource utilisation and specific treatments (e.g. cardiovascular support, targeted temperature management) will be collected using bespoke trial data collection forms; this information will in turn be validated, and where necessary complemented, using information collected from the Intensive Care Research National Audit Programme (ICNARC); (iii) the National Cardiovascular Outcomes Research (NICOR) datasets; iv) information on subsequent hospital inpatient and day case admissions and outpatient visits will be collected though Hospital Episode Statistics; and (v) trial participants or, where necessary, appropriate proxies will be asked to complete economic questionnaires profiling hospital readmissions and post-discharge health and social community care resource use at each time point of follow-up. For the purposes of a sensitivity analysis that will replicate the economic evaluation from a societal perspective, out-of-pocket expenses, and costs associated with lost productivity will also be measured in the economic questionnaires. Current UK unit costs will be applied to each resource item to value total resource use in each arm of the trial. A per diem cost for each level of hospital care, delineated by level of intensity, will primarily be calculated using national tariffs. The unit costs of community health and social services will largely be derived from national sources([8](#_ENREF_8)), although some calculations from first principles using established accounting methods may also be required. The primary analysis will concentrate on direct intervention and healthcare/PSS costs, whilst wider impact (societal) costs will be included within one of the sensitivity analyses.

# Outcomes

In accordance with NICE guidelines([5](#_ENREF_5)), the primary outcome of the within-trial economic evaluation will be the quality-adjusted life year (QALY). This will allow us to generate incremental cost-effectiveness ratios for adrenaline compared with placebo in the form of incremental cost per QALY gained. Secondary health economic outcomes to be considered will include: (i) incremental cost per additional survivor to 30 days post-cardiac arrest, (ii) incremental costs per additional survivor to hospital discharge and (iii) incremental cost per additional neurologically-intact (mRS score) survivor at hospital discharge.

The QALY is a measure that combines quantity and health-related quality of life lived into a single metric, with one QALY notionally equating to one year of full health. QALYs are generated by combining length and health-related quality of life outcomes using area-under-the-curve approaches ([9](#_ENREF_9)). This requires survival and health-related quality of life data from or on behalf of trial participants for the period covering the within-trial time horizon. Health-related quality of life data will be collected for trial participants (see details below) and converted into health-state utilities measured on a 0-1 scale where 1 indicates to full health, and 0 equals death. Note that some multi-attribute utility scales used to measure health-related quality of life can produce negative utility values, reflecting health-states that are considered worse than death ([10](#_ENREF_10)).

Surviving trial participants or, where necessary, appropriate proxies will be asked to complete the EuroQol EQ-5D-5L ([11](#_ENREF_11)) and SF-12 ([12](#_ENREF_12)) measures using postal questionnaire at the 3 and 6 months assessment points. In addition to follow-up data, baseline health-related quality of life are normally required for trial participants in order to generate QALYs for the period covering the relevant time-horizon. Obtaining baseline quality of life data in critical illness settings can be challenging as patients are normally incapacitated or unable to complete patient reported questionnaires at time of randomisation ([13](#_ENREF_13)). Amongst surviving trial participants, health-related quality of life immediately prior to the cardiac arrest will be assessed through a retrospective recall process ([14](#_ENREF_14)) at the 3 months post-randomisation assessment point. Retrospective recall as a method for obtaining baseline health related quality of life for use in trial-based economic evaluation of interventions in critical illness settings has previously been reported ([13](#_ENREF_13), [15](#_ENREF_15)). Briefly, this involves asking trial participants themselves or, where necessary, appropriate proxies to imagine the patient’s health state in the period immediately prior to the event and complete the preferred measurement instrument as if they would have for the period immediately prior to the event. Sensitivity analyses, informed by methods reported in the broader literature ([13](#_ENREF_13)) for dealing with the lack of baseline health utilities will be conducted, for example, assuming that baseline utility values equal zero reflecting an unconscious state.

Responses to the EQ-5D and SF-12 questionnaires will be converted into multi-attribute utility scores using established algorithms ([16](#_ENREF_16), [17](#_ENREF_17)) from which QALYs can be generated. The EQ-5D is a generic preference based 5-dimensional multi-attribute instrument for measuring health-related quality of life. Currently, there are two versions of the questionnaire: a 3-level version (EQ-5D-3L) first introduced in 1990 by the EuroQoL Group ([18](#_ENREF_18)) and a newer 5-level version (EQ-5D-5L) introduced in 2009 ([19](#_ENREF_19)). Patients in the PARAMEDIC-2 trial will complete 5L version of the EQ-5D which can be converted into health utilities using recently published value set for England ([20](#_ENREF_20)). However, NICE recently released a position statement on use of the EQ-5D-5L valuation set ([21](#_ENREF_21)) advising against the use of the new 5L value set ([20](#_ENREF_20)) until the outcome of ongoing research exploring the impact of adopting the EQ-5D-5L valuation set in the NICE reference case becomes available. The position statement further recommends that during this interim period, EQ-5D-5L responses should be mapped or cross-walked onto the EQ-5D-3L using the Hout et al.’s ([22](#_ENREF_22)) algorithm and then derive the health utilities from the EQ-5D-3L utility scores using the UK value set for the EQ-5D-3L ([23](#_ENREF_23)). Therefore, we initially plan to use the utility values derived from cross-walking the EQ-5D-5L to the EQ-5D-3L using the Hout method to generate QALYs for the base case analysis. If NICE subsequently recommends the adoption of the EQ-5D-5L tariffs before completion of the PARAMEDIC-2 trial, the EQ-5D-5L tariff set will be applied to the EQ-5D-5L health state descriptors. A sensitivity analyses will also be conducted using the new 5L value set for England ([20](#_ENREF_20)) and also utilities generated from the SF-12 using the algorithm of Brazier et al ([24](#_ENREF_24)).

# Data quality and cleaning

All data relevant to the health economics analysis will examined for data quality. All questionnaires will be checked for completeness on return to the trial office. Any questionable data will be queried with trial staff. Occasionally, patients or their proxies may provide information that is not clear or appropriate to the question being asked. For example, patients may write “couple of days” or “5-6 days” to the question: *Immediately following your cardiac arrest how many days did you spend in hospital?* Responses such as these will be handled in accordance with pre-specified rules outlined in the PARAMEDIC-2 Data Entry Guidance document to ensure consistency of data entry. Any further issues not resolved by referring to the data entry instructions will be discussed with the trial health economists with clarification sort from the clinical team if necessary. Agreed line of actions for addressing data quality issues will be documented in the data entry guidance documentation. In some instances, patients and or their proxies will be asked to complete an adapted version of the 3 month questionnaire at the 6 month assessment point because they are lost to follow-up at 3 months. The unique patient identifiers for all such records will be kept to enable further sensitivity analysis to be conducted where these patients will be excluded.

# Missing data

Any missing data items present after the data cleaning stage will be addressed within the health economic analysis strategy. Missing data is a common occurrence within RCTs and it is necessary to address it in a standardised principled manner. Within the health economic literature, trial-based economic evaluations have been subject to particular criticism for failing to use appropriate methods to address missing data ([25](#_ENREF_25)). Descriptive analyses of missing data will be carried out (missing data patterns using graphical tools, association between missing data and baseline variables, association between missing data and outcomes). The results of the descriptive analysis will be discussed by the trial team to infer possible reasons for missing data and inform the assumption about the missing data mechanism. In line with best practice recommendations for analysis of within-trial economic data ([26](#_ENREF_26)), multiple imputation by chain equations implemented through the MICE package ([27](#_ENREF_27)) will be used to handle missing data each assessment point. Multiple imputation (MI) generates a series of datasets with each dataset replacing missing values with sampled values. MI replaces each missing observation with a set of plausible imputed values, taken from the predictive distribution of the missing data given the observed data ([28](#_ENREF_28)). Such methods can handle data assumed missing at random (MAR) and can be modified to handle data assumed missing not at random (MNAR) ([29](#_ENREF_29)). Appropriateness of the MAR assumption will be assessed by comparing the characteristics of patients with and without missing data at each follow-up time point. Imputated data will be generated separately by treatment group as recommended by Faria et al ([30](#_ENREF_30)) using the predictive mean matching method which has the advantage of preserving non-linear relationships and correlations between variables within the data.

# Cost-effectiveness analysis

Cost-effectiveness results for the base case analysis will be obtained by formulating a system of seemingly unrelated regressions for individual-level costs and effects, accounting for the patient-level correlations between the two and adjusting for pre-specified baseline patient characteristics. The covariates to be included in the regressions will be those selected a priori for the adjusted statistical analysis, namely age, sex, time to 1^st^ dose administration, witness, bystander CPR, initial aetiology, initial rhythm and total drug dose. Additionally, we will control for any imbalance in baseline costs and EQ-5D values between the two trial arms by including a covariates for baseline costs and health utilities in the regressions, a practice that is now standard for trial-based economic evaluations ([31](#_ENREF_31)). Failure to account for such an imbalance will inevitably lead to biased cost-effectiveness estimates. Incremental effects and costs of adrenaline treatment compared placebo will be generated from the regressions and presented using incremental cost-effectiveness ratios (ICERs) and cost-effectiveness acceptability curves (CEACs). This accommodates sampling (or stochastic) uncertainty and varying levels of willingness to pay for an additional QALY. Heterogeneity in the trial population will be explored by formulating a net-benefit value for each patient from the observed costs and effects, and then constructing a regression model adjusting for the covariates mentioned above. The magnitude and significance of the coefficients on the interaction between the covariates and the treatment variable should provide an estimate of the cost-effectiveness of adrenaline by sub-group. We will also construct a decision-analytical model to model beyond the parameters of the proposed trial the cost-effectiveness of adrenaline in this clinical population. Survival analysis models will be used to estimate life expectancy with and without adrenaline beyond the time horizon of the trial. Long term estimates of costs and health consequences will be discounted to present values using discount rates recommended for health technology appraisal in the United Kingdom. A series of probabilistic sensitivity analyses will be undertaken to explore the implications of parameter uncertainty on the incremental cost-effectiveness ratios.

Pre-specified subgroup analyses

These would include all subgroup analyses pre-specified in the statistical analyses plan that are appropriate to be undertaken for the economic endpoints of interest.

Pre-specified sensitivity analyses

- Incremental costs per QALY gained using health-related quality of life utility values generated from SF-12/SF-6D tariff system.
- Incremental cost per QALY gained using health-related quality of life weights generated from the new EQ-5D-5L value set for England (Devlin et al 2018) ([20](#_ENREF_20)).
- Assume baseline utility of -0.59 (equivalent to unconscious state on the UK EQ-5D-3L value set ([23](#_ENREF_23))) for all patients at baseline
- Restrict analyses to patients who survived to hospital discharged.
- Cardiac arrest witnessed by versus witnessed by bystander versus not witnessed
- Bystander CPR versus no bystander CPR in bystander witnessed and not witnessed patients
- Type of initial rhythm (shockable (VT/VF) versus non-shockable (PEA/Asystole)
- Aetiology of cardiac arrest (medical versus non-medical)
- Age (≤ 60 years vs > 60 years)
- Time interval from 999 call to EMS arrival (≤ 10 minutes vs > 10 minutes)
- Time interval from EMS arrival to administration of trial drug (≤ 10 minutes vs > 10 minutes)
- Time interval from 999 call to administration of trial drug (≤ 10 minutes vs > 10 minutes).

The time to emergency treatment variables were categorised based on previous studies
reporting that administration of adrenaline within 10 minutes following cardiac arrest is associated with neurologically improved survival outcomes ([32](#_ENREF_32)).

# Result tables

Table 1: Completion rates for health economic outcomes

|  | Completion rates | |
| --- | --- | --- |
| Assessment point and resource category | Adrenaline (n=xxx) | Placebo (n=xxx) |
| *Baseline* |  |  |
| EQ-5D-5L index^1^ | xxx% | xxx% |
| EQ-5D-5L VAS^1^ | xxx% | xxx% |
| 3 *months’ assessment point* |  |  |
| Time spent in hospital immediately following cardiac arrest | xxx% | xxx% |
| Had surgery (i.e. coronary angiography with/without PCI)^2^ immediately following cardiac arrest | xxx% | xxx% |
| Use of hospital based or residential care services since being discharged from hospital following cardiac arrest | xxx% | xxx% |
| Use of community based health and social services since cardiac arrest. this includes any services that are not within the hospital for example, visits to the GP) | xxx% | xxx% |
| Medication use | xxx% | xxx% |
| Special equipment or aids | xxx% | xxx% |
| Additional costs | xxx% | xxx% |
| Benefit payments | xxx% | xxx% |
| EQ-5D-5L index | xxx% | xxx% |
| EQ-5D-5L VAS | xxx% | xxx% |
| SF-12 v2 | xxx% | xxx% |
| *6 month months’ assessment point* |  |  |
| Time spent in hospital immediately following cardiac arrest | xxx% | xxx% |
| Had surgery immediately following cardiac arrest | xxx% | xxx% |
| Use of hospital based or residential care services since being discharged from hospital following cardiac arrest | xxx% | xxx% |
| Use of community based health and social services since cardiac arrest. this includes any services that are not within the hospital for example, visits to the GP) | xxx% | xxx% |
| Medication use | xxx% | xxx% |
| Special equipment or aids | xxx% | xxx% |
| Additional costs | xxx% | xxx% |
| Benefit payments | xxx% | xxx% |
| EQ-5D-5L index | xxx% | xxx% |
| EQ-5D-5L VAS | xxx% | xxx% |
| SF-12 v2 | xxx% | xxx% |
| ^1^Restrospective recall of health-related quality of life (EQ5D-5L) immediately prior to cardiac arrest at the 3 month assessment point  ^2^PCI = Percutaneous Coronary Intervention | | |

Table 2: Patient and or proxy reported of health and social care utilisation during trial follow-up

|  |  | Adrenaline(n=xxxx) | | | Placebo (n=xxxx) | | | Adrenaline versus Placebo | |
| --- | --- | --- | --- | --- | --- | --- | --- | --- | --- |
| Assessment point | Category | % missing | Number of visits, mean (se) | Total number of days, mean (se) | % missing | Number of visits, mean (se) | Total number of days, mean (se) | Mean difference, (bootstrap 95% CI)^1^ | P-value |
| 3 months post randomisation | *Inpatient stay immediately following arrest* |  |  |  |  |  |  |  |  |
|  | Intensive care unit |  |  |  |  |  |  |  |  |
|  | Cardiac care unit |  |  |  |  |  |  |  |  |
|  | General ward |  |  |  |  |  |  |  |  |
|  | Other (please specify) |  |  |  |  |  |  |  |  |
|  | Had surgery (i.e. coronary angiography with/without PCI)^2^ immediately following arrest whilst in hospital |  |  |  |  |  |  |  |  |
|  | Inpatient stay since being discharged from hospital |  |  |  |  |  |  |  |  |
|  | *Hospital outpatient clinic* |  |  |  |  |  |  |  |  |
|  | Cardiology |  |  |  |  |  |  |  |  |
|  | Cardiac ‘rehab’ |  |  |  |  |  |  |  |  |
|  | Surgery |  |  |  |  |  |  |  |  |
|  | Other outpatient |  |  |  |  |  |  |  |  |
|  | Cardiology |  |  |  |  |  |  |  |  |
|  | Other hospital/residential care |  |  |  |  |  |  |  |  |
|  | Hospital accident and emergency department |  |  |  |  |  |  |  |  |
|  | Nursing/residential home |  |  |  |  |  |  |  |  |
|  | Other hospital/residential care |  |  |  |  |  |  |  |  |
|  | Community health and social care |  |  |  |  |  |  |  |  |
|  | GP, surgery visit |  |  |  |  |  |  |  |  |
|  | GP, home visit |  |  |  |  |  |  |  |  |
|  | District nurse/Health visitor |  |  |  |  |  |  |  |  |
|  | Social worker |  |  |  |  |  |  |  |  |
|  | Counsellor |  |  |  |  |  |  |  |  |
|  | Home help or care worker |  |  |  |  |  |  |  |  |
|  | Speech and language therapist |  |  |  |  |  |  |  |  |
|  | Psychiatrist/psychologist |  |  |  |  |  |  |  |  |
|  | Day centre |  |  |  |  |  |  |  |  |
|  | Lunch or social club |  |  |  |  |  |  |  |  |
|  | Food, medicine or laundry delivery service |  |  |  |  |  |  |  |  |
|  | Family or patient support or self-help groups |  |  |  |  |  |  |  |  |
|  | Other community care |  |  |  |  |  |  |  |  |
|  | *Other resource use* |  |  |  |  |  |  |  |  |
|  | Medication use |  |  |  |  |  |  |  |  |
|  | Special equipment and aids |  |  |  |  |  |  |  |  |
| 6 months post randomisation | *Inpatient stay immediately following arrest* |  |  |  |  |  |  |  |  |
|  | Intensive care unit |  |  |  |  |  |  |  |  |
|  | Cardiac care unit |  |  |  |  |  |  |  |  |
|  | General ward |  |  |  |  |  |  |  |  |
|  | Other (please specify) |  |  |  |  |  |  |  |  |
|  | *Had surgery* (i.e. coronary angiography with/without PCI)^2^*immediately following arrest whilst in hospital* |  |  |  |  |  |  |  |  |
|  | *Inpatient stay since being discharged from hospital* |  |  |  |  |  |  |  |  |
|  | *Hospital outpatient clinic* |  |  |  |  |  |  |  |  |
|  | Cardiology |  |  |  |  |  |  |  |  |
|  | Cardiac ‘rehab’ |  |  |  |  |  |  |  |  |
|  | Surgery |  |  |  |  |  |  |  |  |
|  | Other outpatient |  |  |  |  |  |  |  |  |
|  | Cardiology |  |  |  |  |  |  |  |  |
|  | Other hospital/residential care |  |  |  |  |  |  |  |  |
|  | Hospital accident and emergency department |  |  |  |  |  |  |  |  |
|  | Nursing/residential home |  |  |  |  |  |  |  |  |
|  | Other hospital/residential care |  |  |  |  |  |  |  |  |
|  | Community health and social care |  |  |  |  |  |  |  |  |
|  | GP, surgery visit |  |  |  |  |  |  |  |  |
|  | GP, home visit |  |  |  |  |  |  |  |  |
|  | District nurse/Health visitor |  |  |  |  |  |  |  |  |
|  | Social worker |  |  |  |  |  |  |  |  |
|  | Counsellor |  |  |  |  |  |  |  |  |
|  | Home help or care worker |  |  |  |  |  |  |  |  |
|  | Speech and language therapist |  |  |  |  |  |  |  |  |
|  | Psychiatrist/psychologist |  |  |  |  |  |  |  |  |
|  | Day centre |  |  |  |  |  |  |  |  |
|  | Lunch or social club |  |  |  |  |  |  |  |  |
|  | Food, medicine or laundry delivery service |  |  |  |  |  |  |  |  |
|  | Family or patient support or self-help groups |  |  |  |  |  |  |  |  |
|  | Other community care |  |  |  |  |  |  |  |  |
|  | *Other healthcare costs* |  |  |  |  |  |  |  |  |
|  | Medication use |  |  |  |  |  |  |  |  |
|  | Special equipment and aids |  |  |  |  |  |  |  |  |
| ^1^mean difference and 95% CIs for total number of days or number of contacts/visits when number of days is not relevant | | | | | | | | | |

Table 3: Patient and or proxy reported of health and social care costs during trial follow-up

|  |  | Adrenaline (n=xxxx) | | | Placebo (n=xxxx) | | | Adrenaline versus Placebo | |
| --- | --- | --- | --- | --- | --- | --- | --- | --- | --- |
| Assessment point | Category | % missing | Number of visits, mean (se) | Total number of days, mean (se) | % missing | Number of visits, mean (se) | Total number of days, mean (se) | Mean difference, (bootstrap 95% CI)^1^ | P-value |
| 3 months post randomisation | *Inpatient stay immediately following arrest* |  |  |  |  |  |  |  |  |
|  | Intensive care unit |  |  |  |  |  |  |  |  |
|  | Cardiac care unit |  |  |  |  |  |  |  |  |
|  | General ward |  |  |  |  |  |  |  |  |
|  | Other (please specify) |  |  |  |  |  |  |  |  |
|  | Had surgery (i.e. coronary angiography with/without PCI)^2^ immediately following arrest whilst in hospital |  |  |  |  |  |  |  |  |
|  | Inpatient stay since being discharged from hospital |  |  |  |  |  |  |  |  |
|  | Total inpatient costs |  |  |  |  |  |  |  |  |
|  | *Hospital outpatient clinic* |  |  |  |  |  |  |  |  |
|  | Cardiology |  |  |  |  |  |  |  |  |
|  | Cardiac ‘rehab’ |  |  |  |  |  |  |  |  |
|  | Surgery |  |  |  |  |  |  |  |  |
|  | Other outpatient |  |  |  |  |  |  |  |  |
|  | Cardiology |  |  |  |  |  |  |  |  |
|  | Other outpatient attendance |  |  |  |  |  |  |  |  |
|  | Total outpatient costs |  |  |  |  |  |  |  |  |
|  | *Other hospital/residential care* |  |  |  |  |  |  |  |  |
|  | Hospital accident and emergency department |  |  |  |  |  |  |  |  |
|  | Nursing/residential home |  |  |  |  |  |  |  |  |
|  | Other hospital/residential care |  |  |  |  |  |  |  |  |
|  | Total hospital/residential costs |  |  |  |  |  |  |  |  |
|  | *Community health and social care* |  |  |  |  |  |  |  |  |
|  | GP, surgery visit |  |  |  |  |  |  |  |  |
|  | GP, home visit |  |  |  |  |  |  |  |  |
|  | District nurse/Health visitor |  |  |  |  |  |  |  |  |
|  | Social worker |  |  |  |  |  |  |  |  |
|  | Counsellor |  |  |  |  |  |  |  |  |
|  | Home help or care worker |  |  |  |  |  |  |  |  |
|  | Speech and language therapist |  |  |  |  |  |  |  |  |
|  | Psychiatrist/psychologist |  |  |  |  |  |  |  |  |
|  | Day centre |  |  |  |  |  |  |  |  |
|  | Lunch or social club |  |  |  |  |  |  |  |  |
|  | Food, medicine or laundry delivery service |  |  |  |  |  |  |  |  |
|  | Family or patient support or self-help groups |  |  |  |  |  |  |  |  |
|  | Other community care |  |  |  |  |  |  |  |  |
|  | *Other healthcare use* |  |  |  |  |  |  |  |  |
|  | Medication use |  |  |  |  |  |  |  |  |
|  | Special equipment and aids |  |  |  |  |  |  |  |  |
|  | Total other healthcare costs |  |  |  |  |  |  |  |  |
|  | **Total costs at 3 month assessment point** |  |  |  |  |  |  |  |  |
| 6 months post randomisation | *Inpatient stay immediately following arrest* |  |  |  |  |  |  |  |  |
|  | Intensive care unit |  |  |  |  |  |  |  |  |
|  | Cardiac care unit |  |  |  |  |  |  |  |  |
|  | General ward |  |  |  |  |  |  |  |  |
|  | Other (please specify) |  |  |  |  |  |  |  |  |
|  | Had surgery (i.e. coronary angiography with/without PCI)^2^ immediately following arrest whilst in hospital |  |  |  |  |  |  |  |  |
|  | Inpatient stay since being discharged from hospital |  |  |  |  |  |  |  |  |
|  | *Total inpatient costs* |  |  |  |  |  |  |  |  |
|  | *Hospital outpatient clinic* |  |  |  |  |  |  |  |  |
|  | Cardiology |  |  |  |  |  |  |  |  |
|  | Cardiac ‘rehab’ |  |  |  |  |  |  |  |  |
|  | Surgery |  |  |  |  |  |  |  |  |
|  | Other outpatient |  |  |  |  |  |  |  |  |
|  | Cardiology |  |  |  |  |  |  |  |  |
|  | Other outpatients |  |  |  |  |  |  |  |  |
|  | Total outpatient costs |  |  |  |  |  |  |  |  |
|  | *Other hospital/residential care* |  |  |  |  |  |  |  |  |
|  | Hospital accident and emergency department |  |  |  |  |  |  |  |  |
|  | Nursing/residential home |  |  |  |  |  |  |  |  |
|  | Other hospital/residential care |  |  |  |  |  |  |  |  |
|  | Total hospital/residential costs |  |  |  |  |  |  |  |  |
|  | *Community health and social care* |  |  |  |  |  |  |  |  |
|  | GP, surgery visit |  |  |  |  |  |  |  |  |
|  | GP, home visit |  |  |  |  |  |  |  |  |
|  | District nurse/Health visitor |  |  |  |  |  |  |  |  |
|  | Social worker |  |  |  |  |  |  |  |  |
|  | Counsellor |  |  |  |  |  |  |  |  |
|  | Home help or care worker |  |  |  |  |  |  |  |  |
|  | Speech and language therapist |  |  |  |  |  |  |  |  |
|  | Psychiatrist/psychologist |  |  |  |  |  |  |  |  |
|  | Day centre |  |  |  |  |  |  |  |  |
|  | Lunch or social club |  |  |  |  |  |  |  |  |
|  | Food, medicine or laundry delivery service |  |  |  |  |  |  |  |  |
|  | Family or patient support or self-help groups |  |  |  |  |  |  |  |  |
|  | Other community care |  |  |  |  |  |  |  |  |
|  | Total community care costs |  |  |  |  |  |  |  |  |
|  | *Other healthcare costs* |  |  |  |  |  |  |  |  |
|  | Medication use |  |  |  |  |  |  |  |  |
|  | Special equipment and aids |  |  |  |  |  |  |  |  |
|  | Total other healthcare costs |  |  |  |  |  |  |  |  |
|  | Total costs at 6 months assessment point |  |  |  |  |  |  |  |  |
|  | **Total costs over 6 months of follow-up** |  |  |  |  |  |  |  |  |
| ^1^mean difference and 95% CIs for total number of days or number of contacts/visits when number of days is not relevant | | | | | | | | | |

Table 4: Patient and or proxy reported additional costs incurred during trial follow-up

|  |  | Adrenaline(n=xxxx) | | | Placebo (n=xxxx) | | | Adrenaline versus Placebo | |
| --- | --- | --- | --- | --- | --- | --- | --- | --- | --- |
| Assessment point | Category | % missing | Number of visits, mean (se) | Total number of days, mean (se) | % missing | Number of visits, mean (se) | Total number of days, mean (se) | Mean difference, (bootstrap 95% CI)^1^ | P-value |
| 3 months post randomisation | Travel costs (e.g. bus fares) |  |  |  |  |  |  |  |  |
|  | Child care costs |  |  |  |  |  |  |  |  |
|  | Income lost |  |  |  |  |  |  |  |  |
|  | Cost of help with housework |  |  |  |  |  |  |  |  |
|  | Cost of laundry services |  |  |  |  |  |  |  |  |
|  | Other additional costs |  |  |  |  |  |  |  |  |
|  | Total additional costs at 3 months assessment point |  |  |  |  |  |  |  |  |
| 6 months post randomisation | Travel costs (e.g. bus fares) |  |  |  |  |  |  |  |  |
|  | Child care costs |  |  |  |  |  |  |  |  |
|  | Income lost |  |  |  |  |  |  |  |  |
|  | Cost of help with housework |  |  |  |  |  |  |  |  |
|  | Cost of laundry services |  |  |  |  |  |  |  |  |
|  | Other additional costs |  |  |  |  |  |  |  |  |
|  | Total additional costs at 6 month assessment point |  |  |  |  |  |  |  |  |
|  | **Total additional costs over 6 months of follow-up** |  |  |  |  |  |  |  |  |
| ^1^mean difference and 95% CIs for total number of days or number of contacts/visits when number of days is not relevant | | | | | | | | | |

Table 5: Sources of unit costs information

| Category | Currency code | Unit cost | Source |
| --- | --- | --- | --- |
| *Inpatient stay* |  |  | e.g.  Reference costs - main schedules (2016) |
| Intensive care unit |  |  |  |
| Cardiac unit |  |  |  |
| General ward |  |  |  |
| Other |  |  |  |
| Other |  |  |  |
| Other |  |  |  |
| *Surgery costs* |  |  |  |
| Stent (x1) |  |  |  |
| Stent (x2) |  |  |  |
| Stent(x3 or more ) |  |  |  |
| Pacemaker |  |  |  |
| ICD |  |  |  |
| PPCI |  |  |  |
| CABG |  |  |  |
| DVT surgery |  |  |  |
| Chest drain surgery |  |  |  |
| CABG, Aortic valve replacement, Mitral valve repair |  |  |  |
| Tracheostomy |  |  |  |
| Trach + PEG |  |  |  |
| Angiogram |  |  |  |
| *Hospital inpatient and residential care after discharge*** |  |  |  |
| Inpatient stay |  |  |  |
| Accident and Emergency |  |  |  |
| Nursing/residential home |  |  |  |
| *Outpatient clinic (per contact)* |  |  |  |
| General surgery | 100 | £130.06 |  |
| Urology | 101 | £105.19 |  |
| Haematology | 303 | £160.58 |  |
| ENT | 120 | £96.87 |  |
| Diabetes | 307 | £159.31 |  |
| Cardiology | 320 | £127.67 |  |
| Neurology | 400 | £175.6 |  |
| Eye Clinic | 460 | £63.46 |  |
| Physiotherapy | 650 | £48.33 |  |
| Medical oncology | 370 | £151.12 |  |
| Rehabilitation unit | VC01Z, VC02Z & VC03Z | £216.11 |  |
| Blood test | DAPS05 | £3 |  |
| *Community health and social care (cost per minute)* |  |  | Community health professional (band 7) cost per hr = £52 (PSSRU 2016, section 9)  £50 per hour (PSSRU, 2014, page 51) updated to 2016 prices using  Nurse (band 7) cost per hour = £52 (PSSRU, 2016, section 10.1)  Practice nurse cost per hour (with qualifications) = £43 (PSSRU 2016, section 10.2)  GP home visits last 11.4 minutes (PSSRU 2015, Table 10.8a). Added cost of 2.18 minutes of GP time for every home visits  cost per surgery consultation lasting 9.22 minutes = £36 (PSSRU 2016, Table 10.3b)  Nurse (band 3) cost per hour = £24 (PSSRU, 2016, section 10.1) |
| GP surgery |  | £3.9 |  |
| GP home visit |  | £3.9 |  |
| District nurse/ health visitor |  | £0.87 |  |
| Social worker |  |  |  |
| Counsellor |  | £0.85 |  |
| Home care worker |  |  |  |
| Speech therapist |  | £0.87 |  |
| Psychology/psychiatry |  | £0.87 |  |
| Day centre |  |  |  |
| Meals on wheels |  |  |  |
| Medicine or laundry delivery service |  |  |  |
| Support or self-help groups |  |  |  |
| *Special equipment* |  |  |  |
| Bed guard |  |  |  |
| ICD with telemetry (remote monitoring) |  |  |  |
| Chair lift |  |  |  |
| Chairs |  |  |  |
| Climate Line Air BPAP/CPAP |  |  |  |
| Commode |  |  |  |
| Crutches |  |  |  |
| Hand rails |  |  |  |
| Heart monitor |  |  |  |
| Hoist |  |  |  |
| Hospital Bed |  |  |  |
| Magnifying glass |  |  |  |
| PEG feed pump |  |  |  |
| Perch Stool |  |  |  |
| Pressure mattress |  |  |  |
| Raised toilet seat |  |  |  |
| Remote cardiac monitor |  |  |  |
| Tilt table |  |  |  |
| Tracker device |  |  |  |
| Urine bottles |  |  |  |
| Walking frame |  |  |  |
| Walking stick |  |  |  |
| Wall rails |  |  |  |
| CABG = Coronary Artery Bypass Grafting  ICD = Implantable cardioverter defibrillator  PPCI = Primary Percutaneous coronary intervention  Trach+PEG = Combined tracheostomy and percutaneous endoscopic gastrostomy | | | |

Table 6: Total economic costs

| Costing perspective and list of included cost categories | Adrenaline (n=xxxx) | | |  | Placebo (n=xxxx) | | |  | Adrenaline versus Placebo | |
| --- | --- | --- | --- | --- | --- | --- | --- | --- | --- | --- |
|  | % missing | % zero costs | Mean (SE), £ |  | % missing | % zero costs | Mean (SE), £ |  | Mean difference (bootstrap 95% CI), £ | P-value^2^ |
| *NHS/PSS perspective* |  |  |  |  |  |  |  |  |  |  |
| Treatment costs |  |  |  |  |  |  |  |  |  |  |
| Follow-up costs |  |  |  |  |  |  |  |  |  |  |
| Total NHS/PSS costs |  |  |  |  |  |  |  |  |  |  |
| *Societal perspective* |  |  |  |  |  |  |  |  |  |  |
| Treatment costs |  |  |  |  |  |  |  |  |  |  |
| Follow-up costs (NHS/PSS) |  |  |  |  |  |  |  |  |  |  |
| Follow-up costs (non-NHS/PSS) |  |  |  |  |  |  |  |  |  |  |
| Total societal costs |  |  |  |  |  |  |  |  |  |  |
| ^1^Confidence intervals obtained by bootstrap percentile method ^2^Two-sided p-values obtained by counting the proportion of bootstrap replicates in which the mean cost-difference is positive, multiplied by 2 and take a minimum | | | | | | | | | | |

Table 7: Summary of EQ5D-5L responses

|  | EQ-5D dimension/ response | Adrenaline (n=xxxx) | Placebo (n=xxxx^)^ | p-value^1^ |
| --- | --- | --- | --- | --- |
| *Baseline* | *Mobility* |  |  |  |
|  | No problems |  |  |  |
|  | Slight problems |  |  |  |
|  | Moderate problems |  |  |  |
|  | Severe problems |  |  |  |
|  | Unable to walk |  |  |  |
|  | Missing |  |  |  |
|  | *Self-care* |  |  |  |
|  | No problems |  |  |  |
|  | Slight problems |  |  |  |
|  | Moderate problems |  |  |  |
|  | Severe problems |  |  |  |
|  | Unable to wash/dress |  |  |  |
|  | Missing |  |  |  |
|  | *Usual activities* |  |  |  |
|  | No problems |  |  |  |
|  | Slight problems |  |  |  |
|  | Moderate problems |  |  |  |
|  | Severe problems |  |  |  |
|  | Unable to do usual activities |  |  |  |
|  | Missing |  |  |  |
|  | *Pain and discomfort* |  |  |  |
|  | No problems |  |  |  |
|  | Slight problems |  |  |  |
|  | Moderate problems |  |  |  |
|  | Severe problems |  |  |  |
|  | Extreme pain and discomfort |  |  |  |
|  | Missing |  |  |  |
|  | *Anxiety and depression* |  |  |  |
|  | No problems |  |  |  |
|  | Slight problems |  |  |  |
|  | Moderate problems |  |  |  |
|  | Severe problems |  |  |  |
|  | Extremely anxious/depressed |  |  |  |
|  | Missing |  |  |  |
| *3 months assessment point* | *Mobility* |  |  |  |
|  | No problems |  |  |  |
|  | Slight problems |  |  |  |
|  | Moderate problems |  |  |  |
|  | Severe problems |  |  |  |
|  | Unable to walk |  |  |  |
|  | Missing |  |  |  |
|  | *Self-care* |  |  |  |
|  | No problems |  |  |  |
|  | Slight problems |  |  |  |
|  | Moderate problems |  |  |  |
|  | Severe problems |  |  |  |
|  | Unable to wash/dress |  |  |  |
|  | Missing |  |  |  |
|  | *Usual activities* |  |  |  |
|  | No problems |  |  |  |
|  | Slight problems |  |  |  |
|  | Moderate problems |  |  |  |
|  | Severe problems |  |  |  |
|  | Unable to do usual activities |  |  |  |
|  | Missing |  |  |  |
|  | *Pain and discomfort* |  |  |  |
|  | No problems |  |  |  |
|  | Slight problems |  |  |  |
|  | Moderate problems |  |  |  |
|  | Severe problems |  |  |  |
|  | Extreme pain and discomfort |  |  |  |
|  | Missing |  |  |  |
|  | *Anxiety and depression* |  |  |  |
|  | No problems |  |  |  |
|  | Slight problems |  |  |  |
|  | Moderate problems |  |  |  |
|  | Severe problems |  |  |  |
|  | Extremely anxious/depressed |  |  |  |
|  | Missing |  |  |  |
| *6 months assessment point* | *Mobility* |  |  |  |
|  | No problems |  |  |  |
|  | Slight problems |  |  |  |
|  | Moderate problems |  |  |  |
|  | Severe problems |  |  |  |
|  | Unable to walk |  |  |  |
|  | Missing |  |  |  |
|  | *Self-care (12 months)* |  |  |  |
|  | No problems |  |  |  |
|  | Slight problems |  |  |  |
|  | Moderate problems |  |  |  |
|  | Severe problems |  |  |  |
|  | Unable to wash/dress |  |  |  |
|  | Missing |  |  |  |
|  | *Usual activities (12 months)* |  |  |  |
|  | No problems |  |  |  |
|  | Slight problems |  |  |  |
|  | Moderate problems |  |  |  |
|  | Severe problems |  |  |  |
|  | Unable to do usual activities |  |  |  |
|  | Missing |  |  |  |
|  | *Pain and discomfort (12 months)* |  |  |  |
|  | No problems |  |  |  |
|  | Slight problems |  |  |  |
|  | Moderate problems |  |  |  |
|  | Severe problems |  |  |  |
|  | Extreme pain and discomfort |  |  |  |
|  | Missing |  |  |  |
|  | *Anxiety and depression (12 months)* |  |  |  |
|  | No problems |  |  |  |
|  | Slight problems |  |  |  |
|  | Moderate problems |  |  |  |
|  | Severe problems |  |  |  |
|  | Extremely anxious/depressed |  |  |  |
|  | Missing |  |  |  |
| ^1^P-values were generated from chi-squared tests for differences in sub-optimal levels of function for each dimension where responses indicating no functional impairment were categorised as optimal and responses indicating any functional impairment were categorised as sub-optimal. | | | | |

Table 8: SF-12 v2 responses

| Assessment point | Response | Adrenaline (n=xxxx) | Placebo (N=xxxx) | P-value^1^ |
| --- | --- | --- | --- | --- |
| *3 month post- randomisation* | *General health* |  |  |  |
|  | Excellent |  |  |  |
|  | Very good |  |  |  |
|  | Good |  |  |  |
|  | Fair |  |  |  |
|  | Poor |  |  |  |
|  | Missing |  |  |  |
|  | *Moderate activities* |  |  |  |
|  | Yes, limited a lot |  |  |  |
|  | Yes, limited a little |  |  |  |
|  | No, not limited at all |  |  |  |
|  | Missing |  |  |  |
|  | *Climbing stairs* |  |  |  |
|  | Yes, limited a lot |  |  |  |
|  | Yes, limited a little |  |  |  |
|  | No, not limited at all |  |  |  |
|  | Missing |  |  |  |
|  | *Accomplished less physically* |  |  |  |
|  | All of the time |  |  |  |
|  | Most of the time |  |  |  |
|  | Some of the time |  |  |  |
|  | A little of the time |  |  |  |
|  | None of the time |  |  |  |
|  | Missing |  |  |  |
|  | *Limited physically* |  |  |  |
|  | All of the time |  |  |  |
|  | Most of the time |  |  |  |
|  | Some of the time |  |  |  |
|  | A little of the time |  |  |  |
|  | None of the time |  |  |  |
|  | Missing |  |  |  |
|  | *Did less Work emotional* |  |  |  |
|  | All of the time |  |  |  |
|  | Most of the time |  |  |  |
|  | Some of the time |  |  |  |
|  | A little of the time |  |  |  |
|  | None of the time |  |  |  |
|  | Missing |  |  |  |
|  | *Accomplished less emotionally* |  |  |  |
|  | All of the time |  |  |  |
|  | Most of the time |  |  |  |
|  | Some of the time |  |  |  |
|  | A little of the time |  |  |  |
|  | None of the time |  |  |  |
|  | Missing |  |  |  |
|  | *Pain* |  |  |  |
|  | Not at all |  |  |  |
|  | A little bit |  |  |  |
|  | Moderately |  |  |  |
|  | Quite a bit |  |  |  |
|  | Extremely |  |  |  |
|  | Missing |  |  |  |
|  | *Calm* |  |  |  |
|  | All the time |  |  |  |
|  | Most of the time |  |  |  |
|  | A good bit of the time |  |  |  |
|  | Some of the time |  |  |  |
|  | A little bit of the time |  |  |  |
|  | None of the time |  |  |  |
|  | Missing |  |  |  |
|  | *Energy* |  |  |  |
|  | All of the time |  |  |  |
|  | Most of the time |  |  |  |
|  | A good bit of the time |  |  |  |
|  | Some of the time |  |  |  |
|  | A little bit of the time |  |  |  |
|  | None of the time |  |  |  |
|  | Missing |  |  |  |
|  | *Feeling down hearted* |  |  |  |
|  | All the time |  |  |  |
|  | Most of the time |  |  |  |
|  | A good bit of the time |  |  |  |
|  | Some of the time |  |  |  |
|  | A little bit of the time |  |  |  |
|  | None of the time |  |  |  |
|  | Missing |  |  |  |
|  | *Social activities* |  |  |  |
|  | All the time |  |  |  |
|  | Most of the time |  |  |  |
|  | A good bit of the time |  |  |  |
|  | Some of the time |  |  |  |
|  | A little bit of the time |  |  |  |
|  | None of the time |  |  |  |
|  | Missing |  |  |  |
| *6 months post randomisation* | *General health* |  |  |  |
|  | Excellent |  |  |  |
|  | Very good |  |  |  |
|  | Good |  |  |  |
|  | Fair |  |  |  |
|  | Poor |  |  |  |
|  | Missing |  |  |  |
|  | *Moderate activities* |  |  |  |
|  | Yes, limited a lot |  |  |  |
|  | Yes, limited a little |  |  |  |
|  | No, not limited at all |  |  |  |
|  | Missing |  |  |  |
|  | *Climbing stairs* |  |  |  |
|  | Yes, limited a lot |  |  |  |
|  | Yes, limited a little |  |  |  |
|  | No, not limited at all |  |  |  |
|  | Missing |  |  |  |
|  | *Accomplished less physically* |  |  |  |
|  | All of the time |  |  |  |
|  | Most of the time |  |  |  |
|  | Some of the time |  |  |  |
|  | A little of the time |  |  |  |
|  | None of the time |  |  |  |
|  | Missing |  |  |  |
|  | *Limited physically* |  |  |  |
|  | All of the time |  |  |  |
|  | Most of the time |  |  |  |
|  | Some of the time |  |  |  |
|  | A little of the time |  |  |  |
|  | None of the time |  |  |  |
|  | Missing |  |  |  |
|  | *Did less Work emotional* |  |  |  |
|  | All of the time |  |  |  |
|  | Most of the time |  |  |  |
|  | Some of the time |  |  |  |
|  | A little of the time |  |  |  |
|  | None of the time |  |  |  |
|  | Missing |  |  |  |
|  | *Accomplished less emotionally* |  |  |  |
|  | All of the time |  |  |  |
|  | Most of the time |  |  |  |
|  | Some of the time |  |  |  |
|  | A little of the time |  |  |  |
|  | None of the time |  |  |  |
|  | Missing |  |  |  |
|  | *Pain* |  |  |  |
|  | Not at all |  |  |  |
|  | A little bit |  |  |  |
|  | Moderately |  |  |  |
|  | Quite a bit |  |  |  |
|  | Extremely |  |  |  |
|  | Missing |  |  |  |
|  | *Calm* |  |  |  |
|  | All the time |  |  |  |
|  | Most of the time |  |  |  |
|  | A good bit of the time |  |  |  |
|  | Some of the time |  |  |  |
|  | A little bit of the time |  |  |  |
|  | None of the time |  |  |  |
|  | Missing |  |  |  |
|  | *Energy* |  |  |  |
|  | All the time |  |  |  |
|  | Most of the time |  |  |  |
|  | A good bit of the time |  |  |  |
|  | Some of the time |  |  |  |
|  | A little bit of the time |  |  |  |
|  | None of the time |  |  |  |
|  | Missing |  |  |  |
|  | *Feeling down hearted* |  |  |  |
|  | All the time |  |  |  |
|  | Most of the time |  |  |  |
|  | A good bit of the time |  |  |  |
|  | Some of the time |  |  |  |
|  | A little bit of the time |  |  |  |
|  | None of the time |  |  |  |
|  | Missing |  |  |  |
|  | *Social activities* |  |  |  |
|  | All the time |  |  |  |
|  | Most of the time |  |  |  |
|  | A good bit of the time |  |  |  |
|  | Some of the time |  |  |  |
|  | A little bit of the time |  |  |  |
|  | None of the time |  |  |  |
|  | Missing |  |  |  |
| ^1^P-values were generated from chi-squared tests for differences in sub-optimal levels of function for each dimension where responses indicating no functional impairment were categorised as optimal and responses indicating any functional impairment were categorised as sub-optimal. | | | | |

Table 9: Summary of health-related quality of life (utility) scores generated from EQ-5D-5L and SF-12 v2 instruments

|  | Adrenaline | | |  | Placebo | | |  | Adrenaline versus Placebo | |
| --- | --- | --- | --- | --- | --- | --- | --- | --- | --- | --- |
| Outcomes | N | % missing | Mean (SE) |  | N | % missing | Mean (SE) |  | Mean difference (95% CI) | P-value |
| *EQ-5D-5L to 3L cross walk^1^* |  |  |  |  |  |  |  |  |  |  |
| Baseline^2^ | xxxx |  |  |  | xxxx |  |  |  |  |  |
| 3 months | xxxx |  |  |  | xxxx |  |  |  |  |  |
| 6 months | xxxx |  |  |  | xxxx |  |  |  |  |  |
| *EQ-5D-5L (new UK tariff)^3^* |  |  |  |  |  |  |  |  |  |  |
| Baseline^2^ | xxxx |  |  |  | xxxx |  |  |  |  |  |
| 6 months | xxxx |  |  |  | xxxx |  |  |  |  |  |
| 12 months | xxxx |  |  |  | xxxx |  |  |  |  |  |
| *SF-12 (SF-6D UK tariff)* |  |  |  |  |  |  |  |  |  |  |
| Baseline^2^ | - |  |  |  | - |  |  |  |  |  |
| 3 months | xxxx |  |  |  | xxxx |  |  |  |  |  |
| 6 months | xxxx |  |  |  | xxxx |  |  |  |  |  |
| *EQ-5D-5L VAS* |  |  |  |  |  |  |  |  |  |  |
| Baseline^2^ | xxxx |  |  |  | xxxx |  |  |  |  |  |
| 3 months | xxxx |  |  |  | xxxx |  |  |  |  |  |
| 6 months | xxxx |  |  |  | xxxx |  |  |  |  |  |
| ^1^The EQ-5D-5L cross-walk utility values were derived using the interim 5L to 3L cross-walk tariffs for the UK ([22](#_ENREF_22))  ^2^Baseline health-related quality of life was collected using the EQ-5D-5L only by retrospective recall at the 3 month assessment point  ^3^New EQ-5D-5L value set for England ([20](#_ENREF_20)) | | | | | | | | | | |

Table 10: Unadjusted estimates of Quality-Adjusted Life Years (QALYs) accrued over 6 months of follow-up

|  | Adrenaline | | |  | Placebo | | |  | Adrenaline versus  Placebo | |
| --- | --- | --- | --- | --- | --- | --- | --- | --- | --- | --- |
| Outcome measure | N | % missing | Mean (SE) |  | N | % missing | Mean (SE) |  | Mean difference (95% CI) | P-value |
| EQ-5D-5L cross-walk tariff | xxxx |  |  |  | xxxx |  |  |  |  |  |
| EQ-5D-5L (New 5L tariff for England) | xxxx |  |  |  | xxxx |  |  |  |  |  |
| SF-12 (SF-6D tariff)^1^ | xxxx |  |  |  | xxxx |  |  |  |  |  |
| QALYs were generated by assuming that baseline utility values measured by the SF-12 (SF-6D) instrument was zero for all patients as this data was not collected for the SF-12 instrument | | | | | | | | | | |

Table 11: Cost-effectiveness results for the within-trial economic analysis

|  | Cost-effectiveness outcomes | | |  | Probability Adrenaline is cost-effective at cost-effectiveness threshold of | | |
| --- | --- | --- | --- | --- | --- | --- | --- |
| Description | Mean incremental costs (95% CI), £ | Mean incremental QALYs (95% CI) | ICER^4^ |  | £13,000 per QALY | £20,000 per QALY | £30,000 per QALY |
| *Base case analysis^1^* |  |  |  |  |  |  |  |
| *Sensitivity analyses* |  |  |  |  |  |  |  |
| Unadjusted analysis |  |  |  |  |  |  |  |
| Complete case analysis |  |  |  |  |  |  |  |
| Per protocol sample |  |  |  |  |  |  |  |
| SF-12/SF-6D |  |  |  |  |  |  |  |
| *Sub-groups* |  |  |  |  |  |  |  |
| Cardiac arrest witnessed by |  |  |  |  |  |  |  |
| Crew/public bystander |  |  |  |  |  |  |  |
| Not witnessed |  |  |  |  |  |  |  |
| Bystander CPR |  |  |  |  |  |  |  |
| Yes |  |  |  |  |  |  |  |
| No |  |  |  |  |  |  |  |
| Type of initial rhythm |  |  |  |  |  |  |  |
| VT/VF |  |  |  |  |  |  |  |
| PEA/Asystole |  |  |  |  |  |  |  |
| Aetiology of cardiac arrest |  |  |  |  |  |  |  |
| Presumed cardiac medical |  |  |  |  |  |  |  |
| Non-cardiac medical |  |  |  |  |  |  |  |
| Time from 999 call to administration of trial drug |  |  |  |  |  |  |  |
| Time category 1 |  |  |  |  |  |  |  |
| Time category 2 |  |  |  |  |  |  |  |
| Time interval from EMS arrival to administration of trial drug |  |  |  |  |  |  |  |
| ≤10 minutes |  |  |  |  |  |  |  |
| >10 minutes |  |  |  |  |  |  |  |
| Time interval from EMS arrival to administration of trial drug |  |  |  |  |  |  |  |
| ≤10 minutes |  |  |  |  |  |  |  |
| >10 minutes |  |  |  |  |  |  |  |
| Age |  |  |  |  |  |  |  |
| ≤60 years |  |  |  |  |  |  |  |
| >60 years |  |  |  |  |  |  |  |
| ICER = Incremental cost-effectiveness ratio; CI = confidence interval  ^1^Adjusted for treatment allocation, age, sex, time to 1^st^ dose administration, witness, bystander CPR, initial aetiology, initial rhythm and total drug dose, baseline health-related quality of life and baseline costs | | | | | | | |

Table 12: Results of economic evaluation expressed in terms of cost per additional survival to 30 days post-cardiac rest and cost per additional neurologically-intact survivor

|  | Cost-effectiveness outcomes | | |  | Probability Adrenaline is cost-effective at cost-effectiveness threshold of | | |
| --- | --- | --- | --- | --- | --- | --- | --- |
| Unit of effectiveness | Mean incremental costs (95% CI), £ | Mean incremental effectiveness (95% CI) | ICER^4^ |  | £13,000 per additional unit of effectiveness | £20,000 per additional unit of effectiveness | £30,000 per additional unit of effectiveness |
| Survival to 30 days post-cardiac arrest |  |  |  |  |  |  |  |
| Survival to hospital discharged |  |  |  |  |  |  |  |
| Neurologically-intact survivor (mRS) at hospital discharge |  |  |  |  |  |  |  |
| ICER = Incremental cost-effectiveness ratio; CI = confidence interval  ^1^Adjusted for treatment allocation, age, sex, time to 1^st^ dose administration, witness, bystander CPR, initial aetiology, initial rhythm and total drug dose | | | | | | | |

# Reference

1. Safar P. Community-Wide Cardiopulmonary Resuscitation. Journal of the Iowa Medical Society. 1964;54:629-35.

2. Morrison LJ, Deakin CD, Morley PT, Callaway CW, Kerber RE, Kronick SL, et al. Part 8: Advanced Life Support. 2010 International Consensus on Cardiopulmonary Resuscitation and Emergency Cardiovascular Care Science With Treatment Recommendations. 2010;122(16 suppl 2):S345-S421.

3. Soar J, Callaway CW, Aibiki M, Böttiger BW, Brooks SC, Deakin CD, et al. Part 4: Advanced life support. Resuscitation.95:e71-e120.

4. Ramsey S, Willke R, Briggs A, Brown R, Buxton M, Chawla A. Good Research Practices for Cost-Effectiveness Analysis Alongside Clinical Trials: The ISPOR RCT-CEA Task Force Report. Value in Health. 2005;8:521-33.

5. NICE. Guide to the methods of technology appraisal. NICE [Internet]. 2013 12 15. Available from: https://[www.nice.org.uk/process/pmg9/resources/guide-to-the-methods-of-technology-appraisal-2013-pdf-2007975843781](http://www.nice.org.uk/process/pmg9/resources/guide-to-the-methods-of-technology-appraisal-2013-pdf-2007975843781).

6. Sanders GD, Neumann PJ, Basu A, et al. Recommendations for conduct, methodological practices, and reporting of cost-effectiveness analyses: Second panel on cost-effectiveness in health and medicine. JAMA. 2016;316(10):1093-103.

7. Husereau D, Drummond M, Petrou S, Carswell C, Moher D, Greenberg D, et al. Consolidated Health Economic Evaluation Reporting Standards (CHEERS) statement. BMJ : British Medical Journal. 2013;346.

8. Curtis L. Unit costs of health and social care. Canterbury, UK: University of Kent; 2011.

9. Glick HA, Doshi JA, Sonnad SS, Polsky D. Economic Evaluation in Clinical Trials. 2nd ed. Gray A, Briggs A, editors. Oxford: Oxford University Press; 2015.

10. Drummond MF, Sculpher M, Torrance GW, O'Brien B, Stoddart GL. Methods for the Economic Evaluation of Health Care Programmes 3ed. Oxford: Oxford University Press 2005.

11. Herdman M, Gudex C, Lloyd A, Janssen M, Kind P, Parkin D, et al. Development and preliminary testing of the new five-level version of EQ-5D (EQ-5D-5L). Quality of life research : an international journal of quality of life aspects of treatment, care and rehabilitation. 2011;20(10):1727-36.

12. Ware JE. How to Score Version 2 of the SF‐12v2® Health Survey (With a Supplement Documenting SF‐12® Health Survey) Lincoln, RI.: QualityMetric Inc; 2002.

13. Dritsaki M, Achana F, Mason J, Petrou S. Methodological Issues Surrounding the Use of Baseline Health-Related Quality of Life Data to Inform Trial-Based Economic Evaluations of Interventions Within Emergency and Critical Care Settings: A Systematic Literature Review. PharmacoEconomics. 2017:1-15.

14. Cuthbertson BH, Roughton S, Jenkinson D, Maclennan G, Vale L. Quality of life in the five years after intensive care: a cohort study. Crit Care. 2010;14(1):R6.

15. Bøhmer E, Hoffmann P, Abdelnoor M, Arnesen H, Halvorsen S. Efficacy and Safety of Immediate Angioplasty Versus Ischemia-Guided Management After Thrombolysis in Acute Myocardial Infarction in Areas With Very Long Transfer Distances: Results of the NORDISTEMI (NORwegian study on DIstrict treatment of ST-Elevation Myocardial Infarction). Journal of the American College of Cardiology. 2010;55(2):102-10.

16. Dolan P, Roberts J. Modelling valuations for Eq-5d health states: an alternative model using differences in valuations. Med Care. 2002;40(5):442-6.

17. Brazier J, Czoski-Murray C, Roberts J, Brown M, Symonds T, Kelleher C. Estimation of a preference-based index from a condition-specific measure: the King's Health Questionnaire. Medical decision making : an international journal of the Society for Medical Decision Making. 2008;28(1):113-26.

18. EuroQol G. EuroQol--a new facility for the measurement of health-related quality of life. Health Policy. 1990;16(3):199-208.

19. Herdman M, Gudex C, Lloyd A, Janssen M, Kind P, Parkin D, et al. Development and preliminary testing of the new five-level version of EQ-5D (EQ-5D-5L). Quality in Life Research. 2011;20(10):1727-36.

20. Devlin NJ, Shah KK, Feng Y, Mulhern B, van Hout B. Valuing health-related quality of life: An EQ-5D-5L value set for England. Health Econ. 2017:n/a-n/a.

21. National Istitute for Health and Care Excellence. Position statement on use of the EQ-5D-5L valuation set. Available from https://[www.nice.org.uk/Media/Default/About/what-we-do/NICE-guidance/NICE-technology-appraisal-guidance/eq5d5l_nice_position_statement.pdf](http://www.nice.org.uk/Media/Default/About/what-we-do/NICE-guidance/NICE-technology-appraisal-guidance/eq5d5l_nice_position_statement.pdf). Accessed on 28 September 2017. 2017.

22. van Hout B, Janssen MF, Feng Y-S, Kohlmann T, Busschbach J, Golicki D, et al. Interim Scoring for the EQ-5D-5L: Mapping the EQ-5D-5L to EQ-5D-3L Value Sets. Value in Health. 2012;15(5):708-15.

23. Kind P, Dolan P, Gudex C, Williams A. Variations in population health status: results from a United Kingdom national questionnaire survey. Brit Med J. 1998;316(7133):736 - 41.

24. Brazier JE, Roberts J. The Estimation of a Preference-Based Measure of Health From the SF-12. Medical Care. 2004;42(9):851-9.

25. Gomes M, Grieve R, Nixon R, Carpenter J, Thompson SG. Developing appropriate methods for cost-effectiveness analysis of cluster randomized trials. Medical Decision Making. 2012;32(2):350-61.

26. Ramsey SD, Willke RJ, Glick H, Reed SD, Augustovski F, Jonsson B, et al. Cost-Effectiveness Analysis Alongside Clinical Trials II 2014;An ISPOR Good Research Practices Task Force Report. Value in Health.18(2):161-72.

27. Van Buuren S, Groothuis-Oudshoorn K. Mice: Multivariate Imputation by Chained Equations. Journal of Statistical Software, 45, 1-67. . 2011.

28. Sterne JA, White IR, Carlin JB, Spratt M, Royston P, Kenward MG. Multiple imputation for missing data in epidemiological and clinical research: potential and pitfalls. BMJ. 2009;338:2393.

29. Faria R, Gomes M, Epstein D. A Guide to Handling Missing Data in Cost-Effectiveness Analysis Conducted Within Randomised Controlled Trials. PharmacoEconomics. 2014;32:1157-70.

30. Faria R, Gomes M, Epstein D, White IR. A Guide to Handling Missing Data in Cost-Effectiveness Analysis Conducted Within Randomised Controlled Trials. PharmacoEconomics. 2014;32(12):1157-70.

31. Manca A, Hawkins N, Sculpher MJ. Estimating mean QALYs in trial-based cost-effectiveness analysis: the importance of controlling for baseline utility. Health Econ. 2005;14(5):487-96.

32. Kudenchuk PJ. Early epinephrine in out-of-hospital cardiac arrest: Is sooner better than none at all? Resuscitation. 2013;84(7):861-2.
