# Supplementary material for: Cost-effectiveness of adrenaline for out-of-hospital cardiac arrest
Source: Crit Care. 2020 Sep 27;24:579. doi: 10.1186/s13054-020-03271-0 (PMC7520962; doi:10.1186/s13054-020-03271-0)
Supplement: Supplementary file 1 — Additional file 1. PARAMEDIC2 HEALTH ECONOMICS ANALYSIS PLAN. Details of the PARAMEDIC2 health economics analysis plan. [file 13054_2020_3271_MOESM1_ESM.zip › Additional file 1.docx]

Table S1: Unit costs for NHS, non-NHS and personal and social service resource inputs (£ sterling, 2017 prices)

| **Type of resource input** | **Unit cost** | **Unit** | **Source** | **Note** |
| --- | --- | --- | --- | --- |
| ***Emergency response*** |  |  |  |  |
| Emergency ambulance | £8.00 | Minute | PSSRU UC 2008, page 82 | £6.80 (2008) prices inflated to 8.00 (2017 prices) |
| Adrenaline injection | £6.87 | Syringe | BNF 2016/17 | 1mg/10ml (1 in 10,000) dilute solution for injection pre-filled syringes (Martindale Pharmaceuticals Ltd) |
|  |  |  |  |  |
| ***The primary source of the unit cost of hospital services (raw resource use data derived from Hospital Episode Statistics), Mean (SD), range*** | | | | |
| Inpatients (initial) £6,022.47 (20,869.58), 352 to 683,381 |  | Inpatient spell | National Ref costs 2016-17 |  |
| Inpatients (readmission)  £3,804.74 (20,877.28), 211 to 41,151 |  | Inpatient spell | National Ref costs 2016-17 |  |
| Critical care (initial)  £11,289.47 (19,539.52), 348 to 364,247 |  | Inpatient spell | National Ref costs 2016-17 |  |
| Critical care (readmissions)  £13,817.98 (18,635.79), 1,202 to 64,739 |  | Inpatient spell | National Ref costs 2016-17 |  |
| Accident and emergency  £233.79 (93.93), 83 to 1,045 |  | Visit | National Ref costs 2016-17 |  |
| Outpatients (first appointment)  £291.41 (423.15), 32 to 1,714 |  | Attendance | National Ref costs 2016-17 |  |
| Outpatients (follow-up)  £353.95 (423.73), 32 to 5,659 |  | Attendance | National Ref costs 2016-17 |  |
|  |  |  |  |  |
| *Secondary source of unit costs for hospital services (raw resource use data derived from trial case report forms )* | | | | |
| *Inpatient care* |  |  |  |  |
| ED visit | £389.81 | Visit | National Ref costs 2016-17 | Emergency medicine (service code T01A; currency code VB02ZZ) |
| ICU bed-day | £2,114.13 | Bed-day | National Ref costs 2016-17 | Average cost of 0–6 or more organs supported (CCU01 and XC01Z-XC07Z) weighted by activity |
| Cardiac ward bed-day | £305.85 | Bed-day | National Ref costs 2016-17 | Non-elective inpatients; excess bed-days |
|  |  |  |  |  |
| **Secondary (*Outpatient services)*** | | | | |
| Cardiology | £137.07 | Attendance | National Ref costs 2016-17 | Service code 320 |
| Cardiac rehab | £79.70 | Attendance | National Ref costs 2016-17 | Service code 327 |
| Surgery | £141.19 | Attendance | National Ref costs 2016-17 | Service code 100 |
| Neurology | £171.98 | Attendance | National Ref costs 2016-17 | Service code 400 |
| Ophthalmology | £95.15 | Attendance | National Ref costs 2016-17 | Service code 130 |
| Urology | £111.61 | Attendance | National Ref costs 2016-17 | Service code 101 |
| Angiogram | £169.00 | Attendance | National Ref costs 2016-17 | RD32Z Contrast Fluoroscopy Procedures with duration of more than 40 minutes |
| Echocardiogram | £74.00 | Attendance | National Ref costs 2016-17 | RD51A |
| Haematology/oncology | £176.24 | Attendance | National Ref costs 2016-17 | 370 |
| Podiatry | £45.81 | Attendance | National Ref costs 2016-17 | 653 |
| Nursing/residential home | £162.00 | Attendance | PSSRU Unit Costs 2017 | Local authority own-provision residential care for older people (£162/day, page 35) |
| Home carers twice a day | £27.00 | Attendance | PSSRU Unit Costs 2017 | Page 178. Face-to-face: 26 per hour weekday (£27 per day-time weekend, 27 per night-time weekday), assumed an hour of care provided each day @ 27/hour |
| Other outpatient services  Mean (SD), range  £155.78 (244.19), 1 to 2,114 |  |  |  |  |
|  | | |  |  |
| ***Community health and social care services*** | | |  |  |
| Counsellor | £53.00 | Contact | PSSRU Unit Costs 2017 | Community-based professional (band 7) costing 53 per hour (pages 153-155); Average length of surgery consultation (PSSRU Unit Costs 2013, page 54). |
| District nurse | £41.04 | Contact | PSSRU Unit Costs 2013 | The mean average cost for a face-to-face contact in district nursing services for 2012/2013 was 39, inflated to 2017 prices |
| GP, home visit | £38.00 | Contact | PSSRU Unit Costs 2017 | Per patient contact lasting 9.22 minutes including CO2 emissions (page 162) |
| GP, surgery visit | £37.00 | Contact | PSSRU Unit Costs 2017 | Per surgery consultation lasting 9.22 minutes (page 162) |
| GP, telephone consultation | £28.49 | Contact | PSSRU Unit Costs 2017 | Per telephone consultation lasting 7.1 minutes (PSSRU Unit Costs 2013, page 191) |
| Home care worker | £13.50 | Contact | PSSRU Unit Costs 2017 | Page 178. Face-to-face: 26 per hour weekday (£27 per day-time weekend, 27 per night-time weekday, 27 per night-time weekend). Assumed 30 minutes contact at 27 per hour |
| Meals and laundry | £6.94 | One meal | PSSRU Unit Costs 2014 | The average cost per meal on wheels was 6.60 for the local authority in 2012/13 (PSSRU Unit Costs 2014, page 127). Inflated to 2017 prices using the Hospital & community health services (HCHS) Pay and Price index |
| Occupational Therapy | £64.99 | Contact | National Ref costs 2016-17 | Occupational Therapy (service code 951) |
| Physiotherapy | £48.00 | Contact | National Ref costs 2016-17 | Service code 650 |
| Practice Nurse | £10.85 | Contact | PSSRU Unit Costs 2017 | Practice nurse hourly costs including qualifications 42 (page 160); Duration of contact 15.5 minutes (PSSRU Unit Costs 2013, page 188) |
| Psychiatrist | £84.57 | Contact | National Ref costs 2016-17 | Liaison Psychiatry (service code 722) |
| Psychology | £168.65 | Contact | National Ref costs 2016-17 | Clinical Psychology (service code 656) |
| Lunch or social club | £6.94 | One meal | PSSRU Unit Costs 2014 | The average cost per meal on wheels was 6.60 for the local authority in 2012/13 (PSSRU Unit Costs 2014, page 127). Inflated to 2017 prices using the Hospital & community health services (HCHS) Pay and Price index |
| Social worker | £43.07 | Contact | PSSRU Unit Costs 2017 | Social worker (adult services) with qualifications cost 59 per hour, page 174; assumed 73% of time is spent on client-related activities (PSSRU Unit Costs 2017, page 174) including direct contact (includes travel) |
| Speech therapy | £96.52 | Contact | National Ref costs 2016-17 | Service code 652 |
| Other primary care services, Mean (SD), range  £58.98 (19.43), 21 to 98 |  |  |  |  |
| **Additional costs**^1^ over 6 months, N Mean (SD), range for adrenaline group | 61, £3,634 (£6,590.44), £0.93 to £31,400 | |  |  |
| **Additional costs over 6 months,** N Mean (SD), range for adrenaline group | 40, £2,468 (£3,805.61), £0.20 to £16,040 | |  |  |
| ^1^Additional non-health and social care costs reported by patients or their proxies. Include items such as childcare costs, purchase of equipment aids/adaptations, over-the-counter medication, laundry, hospital parking charges and lost income. | | | | |
