# Supplementary material for: Cost-effectiveness of adrenaline for out-of-hospital cardiac arrest
Source: Crit Care. 2020 Sep 27;24:579. doi: 10.1186/s13054-020-03271-0 (PMC7520962; doi:10.1186/s13054-020-03271-0)
Supplement: Supplementary file 3 — Additional file 3. Pre-specified and post-hoc sensitivity analyses exploring varying features of the within-trial economic evaluation on the cost-effectiveness results. List of sensitivity analyses. [file 13054_2020_3271_MOESM3_ESM.zip › 2020-06-27-Additional file 3.docx]

Additional file 3: Pre-specified and post-hoc sensitivity analyses exploring varying features of the within-trial economic evaluation on the cost-effectiveness results

| **Sensitivity analysis** | **Base-case methods/assumptions** | **Changes implemented in sensitivity analyses** |
| --- | --- | --- |
| *Pre-specified sensitivity analyses* | |  |
| SA1 | Adjusted multiple imputation | Unadjusted multiple imputation |
| SA2 | Adjusted multiple imputation | Adjusted complete case analysis |
| SA3 | Adjusted multiple imputation | Unadjusted complete case analysis |
| SA4 | Parameter estimates via seemingly unrelated linear regression | Parameter estimates via linear regression implemented within a bootstrap |
| SA5 | QALYs based on EQ-5D-5L/mRS data | QALYs based on mRS data only |
| SA6 | QALYs derived assuming baseline utility of 0 (equivalent to dead) | QALYs derived assuming baseline utility of -0.042 (equivalent to unconscious health state) |
| SA7 | QALYs based on EQ-5D-5L and mRS data, 6 months NHS/PSS costs | QALYs based on EQ-5D-5L and mRS data, 6 months Societal costs |
| SA8 | 6 month time horizon (QALYs based on EQ-5D-5L and mRS data, NHS/PSS costs) | 12-month time horizon (QALYs based on EQ-5D-5L and mRS data, NHS/PSS costs)^±^ |
| SA9 | QALYs based on EQ-5D-5L and mRS data, 6-month time horizon | QALYs based on mRS data, NHS/PSS costs, 12-month time horizon^±^ |
| SA10 | QALYs based on EQ-5D-5L and mRS data, six-month time horizon | QALYs based on QALYs based on EQ-5D-5L and mRS data, Societal costs, 12-month time horizon^±^ |
| *Post-hoc sensitivity analyses* | |  |
| SA11 | One hour added to emergency response cost calculations to account for stand-down and restock time | Excluded stand down and restock time in emergency response cost calculations |
| SA12 | Accounts for the cost of transporting deceased patients to the nearest hospital or local authority mortuary if the patient died at scene of cardiac arrest and in a public place | Excluded estimated cost of transporting deceased patients to nearest hospital moctuary if patient died at scene of cardiac arrest and in a public place |
| SA13 | Accounts for stand down/re-stock time and transportation costs to nearest mortuary if patient died in a public place in emergency response cost calculations | Excluded stand down/restock time and cost of transporting patients to nearest moctuary in emergency response cost calculations |

SA denotes sensitivity analysis; QALY denotes quality-adjusted life-year; mRS denotes modified Rankin Scale; NHS denotes National Health Service; PSS denotes personal and social services.

^±^ Data between 6 and 12 months post-randomization were drawn from the HES and PEDW databases for hospital costs and linear extrapolations of other economic variables based on data between 3 and 6 months post-randomization.
