# Supplementary material for: Cost-effectiveness of adrenaline for out-of-hospital cardiac arrest
Source: Crit Care. 2020 Sep 27;24:579. doi: 10.1186/s13054-020-03271-0 (PMC7520962; doi:10.1186/s13054-020-03271-0)
Supplement: Supplementary file 4 — Additional file 4. Subgroup analyses conducted that explored potential heterogeneity in the within-trial incremental cost-effectiveness of adrenaline. [file 13054_2020_3271_MOESM4_ESM.zip › 2020-06-27-Additional file 4.docx]

Additional file 4: Subgroup analyses conducted that explored potential heterogeneity in the within-trial incremental cost-effectiveness of adrenaline

| Aetiology of cardiac arrest (medical vs. non-medical) |
| --- |
| Age of patient (≤ 60 years vs. > 60 years) |
| Gender (female vs. male) |
| Time interval from EMS arrival to administration of trial drug (≤ 10 minutes vs. > 10 minutes) among those with a witnessed arrest^±^ |
| Time interval from 999 call to EMS arrival (≤ 10 minutes vs. > 10 minutes) among those with a witnessed arrest^±^ |
| Type of initial rhythm (shockable (Ventricular Tachycardia/ Ventricular Fibrillation) vs. non-shockable (Pulseless Electrical Activity /Asystole)) |
| Cardiac arrest witnessed by paramedic vs. cardiac arrest witnessed by bystander vs. not witnessed |
| Bystander CPR vs. no bystander CPR in bystander witnessed and not witnessed patients |
| ^±^ The time to emergency treatment variables were categorised based on previous studies reporting that administration of adrenaline within 10 minutes following cardiac arrest is associated with neurologically improved survival outcomes |
