# Supplementary material for: Cost-effectiveness of adrenaline for out-of-hospital cardiac arrest
Source: Crit Care. 2020 Sep 27;24:579. doi: 10.1186/s13054-020-03271-0 (PMC7520962; doi:10.1186/s13054-020-03271-0)
Supplement: Supplementary file 4 — Additional file 4. Subgroup analyses conducted that explored potential heterogeneity in the within-trial incremental cost-effectiveness of adrenaline. [file 13054_2020_3271_MOESM4_ESM.zip › Additional file 4.docx]

# Additional File 4: Economic modelling to extrapolate cost-effectiveness beyond trial follow-up and incorporate the indirect effects of organ donation

# Extrapolations of cost-effectiveness beyond the parameters of the PARAMEDIC2 trial

A simple Markov state transition model was built in R statistical programming software^1^ using the package heemod^2^ to extrapolate the within-trial results over the lifetimes of cardiac arrest survivors. The model as shown in as shown in plot A of Figure 1 comprises of four health-states (out of hospital cardiac arrest (OHCA) state, post-OHCA survival with good neurologic function, post-OHCA survival with poor/impaired neurologic function, and death). The cycle length is one-year, and all individuals start in the OHCA state at the incidence of cardiac arrest event. Possible transitions from this state represent one-year survival with good or poor neurological function and death. This implies that the OHCA state captures all the economic costs and benefits in the first year after the cardiac arrest event and so corresponds to the sensitivity analysis in which the within-trial time horizon was extended to cover the first-year post-randomisation period. In PARAMEDIC2, neurological function was assessed at hospital discharge and at 3 months and 6-months post-randomisation. This information was used to categorise post-OHCA survival as good if the MRS assessment closest to the one-year time point after the cardiac arrest event was less than or equal to 3 and poor for scores greater than 3. Probabilities governing these transitions were estimated separately for the adrenaline and placebo groups based on these categorisations and are summarised in the Table A1 of Additional file 4.

Possible transitions beyond the first year after the cardiac arrest event were restricted to movement from the good and poor functional states to death. The probabilities governing these movements were assumed to be independent of treatment allocation and estimated from two sources. First, we estimated the conditional probability of surviving to 12 months given that an individual had survived the first 9 months with good or poor function and converted these estimates to one-year probabilities using standard formulae.^3^ Second, we obtained age and sex-adjusted UK mortality statistics (Office for National Statistics 2018) assuming a starting age of 60 years and 78% probability of being male, reflecting average cohort characteristics of survivors at one-year post randomisation. We used this information to extrapolate survival beyond the first-year post cardiac arrest to a lifetime horizon by assigning individuals a probability of death equivalent to the larger of the two estimates.

We assumed that patients surviving beyond the first year after the cardiac arrest event either died or remained alive in the same functional state at each modelled cycle (functional status cannot improve or deteriorate after first-year post cardiac arrest). The assumption that neurological function cannot improve or deteriorate after the first year post cardiac arrest was made due to a lack of robust data to estimate the between state transitions and is consistent with recent economic models evaluating the cost-effectiveness of intervention in cardiac arrest.^4^

Patients in the OHCA state were assigned cost and utility values obtained from the within-trial sensitivity analysis in which the time horizon was extended to one-year. This covered the full spectrum of costs and QALYs incurred in the first year after the cardiac arrest event adjusted for demographic and clinical characteristics. Costs associated with the good and poor functional states were estimated from resource use estimates covering the 3-6 month post-randomisation period collected for trial participants. Utility values associated with good and poor functional states were collected through the EQ-5D-5L reported by trial participants and stratified by functional status at the 6 month assessment point (Table A1, Additional file 4). Costs generated from resource use covering the 3-6-month period after randomisation were converted to one-year costs assuming that the rate of resource use in this 3-month window is maintained over a period of one-year. Costs and utilities were discounted at 3.5% per annum over the lifetime of cardiac arrest survivors.

Data inputs for the economic modelling to extrapolate cost-effectiing beyond trial follow-up

| **Parameter** | **Mean (SE)** | **Distribution** | **source** |
| --- | --- | --- | --- |
| *Cohort characteristics* |  |  |  |
| Mean age, one-year post cardiac arrest survivors | 60 year |  | PARAMEDIC2 trial data |
| Percentage male among one-year post cardiac arrest survivors | 78% | Beta | PARAMEDIC2 trial data |
|  |  |  |  |
| *Probabilities* |  |  |  |
| 1-year survival with good function (MRS≤3), adrenaline | 0.023 (0.0024) | Multinomial | PARAMEDIC2 trial data |
| 1-year survival with good function (MRS≤3), placebo | 0.019 (0.0021) | Multinomial | PARAMEDIC2 trial data |
| 1-year survival with poor function (MRS>3), adrenaline | 0.005 (0.0011) | Multinomial | PARAMEDIC2 trial data |
| 1-year survival with poor function (MRS>3), placebo | 0.003 (0.0019) | Multinomial | PARAMEDIC2 trial data |
| 3-month mortality given alive with good function at 9 months post arrest | 0.029 (0.013) | Binomial | PARAMEDIC2 trial data |
| 3-month mortality given alive with poor function at 9 months post arrest | 0.061 (0.042) | Binomial | PARAMEDIC2 trial data |
| Annual mortality rate given survival to one-year with good function | 0.111 (0.0457) | Derived | 0.111/ UK mortality 2015 |
| Annual mortality rate given survival to one-year with poor function | 0.215 (0.1213) | Derived | 0.215 /UK mortality 2015 |
|  |  |  |  |
| *Costs (£, 2017 prices)* |  |  |  |
| OHCA, adjusted mean costs, 1-year post-randomization, adrenaline | £1,973 (£663) | Gamma | PARAMEDIC2 trial data |
| OHCA, adjusted mean costs, 1-year post-randomization, placebo | £663 (£563) | Gamma | PARAMEDIC2 trial data |
| Good functional state | £7,907 (£718) | Gamma | PARAMEDIC2 trial data |
| Poor functional state | £24,457 (£2,275) | Gamma | PARAMEDIC2 trial data |
|  |  |  |  |
| *Utilities (EQ-5D-5L)* |  |  |  |
| OHCA, adjusted mean utility, first-year post-randomization, adrenaline | 0.0048 (0.005) | Beta | PARAMEDIC2 trial data |
| OHCA, adjusted mean utility, first-year post-randomization, placebo | 0.0027 (0.0027) | Beta | PARAMEDIC2 trial data |
| Good functional state | 0.707 (0.019) | Beta | PARAMEDIC2 trial data |
| Poor functional state | 0.0982 (0.0511) | Truncated normal | PARAMEDIC2 trial data |

## **Organ donation modelling overview**

A previous economic model of adult lung transplantation^5,6^ was adapted to simulate the impact of adrenaline on the supply of organs for transplantation from deceased donors. We constructed separate models to simulate costs and consequences associated with liver, lung and kidney transplantations over the lifetimes of patients needing a graft. The base case analysis was conducted from the perspective of the UK NHS and Personal Social Services. Costs, valued at 2017 prices, and quality-adjusted life years (QALYs) were calculated over a life-time horizon and discounted at 3.5% per annum to reflect current methodological recommendations from the National Institute of Health and Care Excellence (NICE).^7^

## Transplant model structure

The model was constructed in Microsoft Excel 2016 to simulate pre and post-transplantation survival for individuals on the active transplant waiting list. Figure 1b depicts a simplified version of the original model described for the DEVELOP-UK HTA programme.^5,6^ It contains five health states: waiting list, removed from waiting list, two post-transplantation health states and death. Removal from waiting list and death are absorbing states in that individuals can enter but not leave these states. The cycle length is one year and all individuals start in the waiting list state. At each cycle, individuals can move to post-transplantation upon receiving a transplant, removed from the waiting list, die or remain on the waiting list. The probabilities governing these transitions were obtained from 2-year, 3-year and 5-year post-registration outcome data for new adult liver, lung and kidney registrations in the UK covering the period 1 April 2012 and 31 March 2015.^8-10^ Because a small proportion of individuals remain on the waiting list beyond the second, third and fifth year for which data were available, we allowed individuals to remain on the waiting list up to 10 years from joining the register. We assumed that individuals would not contribute costs and benefits beyond 10 years on the waiting list. Post-transplantation, patients either died or remained alive; however, actual transitions between the post-transplantation states were not implemented. Instead, we assigned an overall mean post-transplantation survival to transplant recipients and partitioned this time between two health states: a first year post-transplantation state to capture first-year post-transplantation survival, healthcare costs and benefits, and a post-transplantation (year>one) health-state to capture survival, costs and benefits beyond year one.

## Waiting list transitions

Waiting list transitions were informed by annual post-registration outcomes data for 296 UK wide adult lung, 904 liver and 2873 kidney only registrations obtained from the 2017-18 NHS Blood and Transplant (NHSBT) activity reports.^8-10^ The data are summarised in Table A2 and show the proportion of patients transplanted, remaining on the waiting list, removed from the list and dying on the list at one, two, three and five years from joining the list. We combined this information with data on number of deceased donor transplants that took place between 1^st^ April 2017 and 31^st^ March 2018 (196 lungs, 950 livers and 2319 kidneys)^8^ to derive annual probabilities of transplantation for each year that an individual remains on the waiting list up to 10 years post-registration. To simplify the calculations, we assumed that the donation and demand for organs has reached equilibrium so that the numbers joining the list each year equalled those leaving it. We further assumed that the probabilities of being removal from the waiting list and death on the waiting list were independent of organ donation and demand. Under these steady state assumptions, an increase in organ donation without concurrent increase in demand would increase the probability of transplantation as more organs become available and vice versa. With these assumptions and NHSBT data, we can derive how organs that become available each year will be distributed among individuals on the waiting list and calculate the probability of transplant under alternative scenarios that impact on organ supply.

Distribution of organs for transplantation

We first derive the distribution of organs that become available for transplantation each year among individuals on the lung register and repeat the process for livers and kidneys. Details of the calculations are given in the sheet labelled ‘Organ supply’ of the respective model coded in Microsoft Excel for each organ. The NHSBT data^9^ suggested that, of the 296 adult lung only registrations in the 2014-15 transplant activity year, 57% had received a transplant within three years of joining the register, 26% had died and 8% were taken off the register over three years (Table A2, Additional file 4). Extending the third year transitions for the 8% who remained on the register beyond year 3 to cover the period from year 4 to year 10 yielded 179 (60%) lung transplantations over 10 years, 80 deaths (27%), 37(12.5%) removals from list and less than 1% remaining on the list beyond year 10 (see ‘Organ supply’ sheet of Microsoft Excel model for lung transplantation). The one, two and three year probabilities of transplant in the NHSBT report^9^ implied that 133 (75%) of the 179 transplantations went to individuals in the first year of joining the list, 27 (15%) to individuals in year two, 9 (5%) to those waiting three years and only 5 (3%) to individuals waiting more than three years. In any transplant activity year, there are a mixture of individuals who stand to benefit for transplantation with varying length of time on the register. Under the steady state assumptions, the distribution of organs over the 10 year waiting list is equivalent to assuming that within any one year, 75% of available organs go to individuals within one year of joining the list, 15% to individuals waiting 2 years and 8% to individuals waiting three or more years (excel model for lung transplantation, row R of sheet labelled ‘Organ supply’).

Applying the above calculations to the 904 adult liver only registrations in the 2014-15 activity year^8^ generated 727 (80.5%) transplantations, 115 (12.8%) deaths on the waiting list and 61 (6.8%) removals from the list over 10 years. The one and two-year probabilities of liver transplant in the NHSBT report^8^ would imply that 615 (85%) of the 729 liver transplantations went to individuals in the first year of joining the list, 81 (11%) to individuals in waiting two years, 23 (3%) to those waiting three years and 8 (2.4%) to individuals waiting more than 3 years (see excel model for liver transplantation, row R of sheet labelled ‘Organ supply’). Similarly, for kidneys, applying the calculations to the 2873 individuals joining the register in the 2012-13 activity year^8^ generated 2277 (79%) transplantations over 10 years, 243 (8%) deaths on the waiting list, 343 (12%) removals from the list and only 10 (0.34%) individuals remaining on the list beyond year 10. Applying the reported one, three and five-year probabilities of kidney transplant implies that 747 (33%) of the 2277 transplants over 10 years went to individuals within one year of registering. A further 890 (39%) received a kidney in the second and third year of registering, 430 (19%) to individuals on the list for four and five years and 209 (9%) to individuals waiting more than five years (excel model for kidney transplantation, row R of sheet labelled ‘Organ supply’).

## Probability of transplant under alternative treatment strategies

In the base-case analysis, the probability of receiving a cardiac arrest donated organ in the adrenaline arm was set at 25% based on analyses of ICNARC data^11^ and an understanding that current UK clinical practice involves adrenaline use in cardiac arrest resuscitation. This equates to 49 out of the 196 lung, 249 out of the 950 liver and 580 out of the 2319 deceased kidney transplantations*^[[1]](#footnote-1)^* that took place in the 2017-18 activity year^7^ assuming no organ wastage. To derive the equivalent numbers of transplantations for the placebo arm, we used information on transplantation rates in the PARAMEDIC2 trial to calculate odds ratio*^[[2]](#footnote-2)^* for a cardiac arrest patient donating a transplanted organ in the adrenaline arm compared with placebo. The trial data suggested that 4 individuals received lungs, 25 received livers and 67 received kidneys donated by cardiac arrest patients from a potential donor pool of 4015 individuals randomised to receive adrenaline. For the placebo group, the equivalent numbers were 3 lung, 16 liver and 43 kidney transplantations from a potential donor pool of 3999 patients. These figures translate to 33% fewer lung, 50% fewer liver and 56% fewer kidney transplantations in the placebo arm compared with adrenaline/current practice (Table A2, Additional file 4). Applying the relative reduction in transplantation rates observed in the PARAMEDIC2 trial to the baseline transplantation rates implies that 37 lung, 166 liver and 372 kidney transplantations each year would take place on average following placebo. Assuming the distribution of transplanted organs is independent of organ donation (i.e. 75% of available lungs, 85% of available livers and 33% of available kidneys would go to individuals within one year of joining the list, and so on regardless of supply side effects and hence treatment allocation), the probability of transplantation will increase as more organs become available and decrease with reduced donation. For the adrenaline arm, the required probabilities will be those estimated by extrapolating observed NHSBT waiting list data over 10 years and ranged from 45% at year one to 60% over 10 years for lungs, 68% at year one to 81% over 10 years for livers and 26% at year one to 79% over 10 years for kidneys. The corresponding probabilities of transplantation for the placebo arm ranged from 34% at year one to 48% over 10 years for lungs, 45% at year one to 55% over 10 years for livers and 17% at year one to 53% over 10 years for kidneys. Details of these calculations are provided in the sheet labelled ‘Organ supply’ of the excel models.

## Post-transplantation survival

Following transplantation, patients were assigned a mean survival time estimated from the literature using a variety of methods. Mean survival following lung transplantation was estimated to be 8.2 years using area-under-the-curve methods described in the DEVELOP-UK report.^5,6^ This was based on risk-adjusted post-lung transplant survival of 78%, 70%, 53% and 34% at one, three, five and ten years based on 2017/18 NHSBT data,^9^ a 19-year post-transplantation survival of 12% and maximum life-expectancy for lung transplant patients of 25 years.^5,6^ Mean survival following liver transplantation was estimated at 32 years based on a median survival time of 22.5 years assuming an exponential survival function and based on analyses of NHSBT data for a cohort of 2702 adult liver graft recipients between 1985 and 2003.^12^ Mean post-transplantation survival for kidneys was estimated at 20.5 years based on analyses of NHSBT data for 12,000 recipients of deceased donor kidney or combined kidney and pancreas transplantation between 2003 and 2012.^13^ The authors^13^ fitted several parametric distributions to extrapolate beyond follow-up time taking into account clinical characteristics of the cohort and used this to predict mean overall survival time. The estimated mean survival values were 12.7 years for a 55-year old male recipient with Type 1 diabetes, 20.5 years for a 50-year old female recipient with glomerulonephritis and 25.7 years for a 50-year old recipient with polycystic kidney disease. We used the 20-year estimate in our base-case analyses and conducted sensitivity analyses using the lower and upper estimates.

## Healthcare and personal social service costs

The analysis considered costs from a UK health and personal social services perspective. These included treatment costs whilst waiting for transplant, organ extraction and preservation activity, cost of surgery and post-transplantation treatment costs. Costs were extracted from our review of the literature to identify data inputs and inflated to 2016-17 prices where necessary using the National Health Service Hospital and Community Health Services Pay and Prices Index, and the Consumer Prices Index.^14^ The data are summarised in Table A2 (Additional file 4). The cost of transplant related activity and post-transplantation costs were taken primarily from the DEVELOP-UK report^5^ for lungs and the ‘Commissioning Transplantation to 2020’ report for livers and kidneys.^15^ Following the approach described DEVELOP-UK study,^5^ we categorised transplantations as standard and non-standard procedures requiring expensive interventions such as Ex-vivo lung perfusion (EVLP) or in situ /ex-vivo machine perfusion (EVMP) to recondition organs prior to transplantation.^15^ The proportion of EVLP/EVMP transplants was set to 10% based on the UK DEVELOP data for lungs and livers (due to lack of liver-specific data)^5,6^ and 20% for kidneys (NICE Technology Appraisal TA165).^16^ The cost of deceased kidney extraction and preservation was assumed to be the same as corresponding costs for liver extraction and preservation due to lack of relevant UK cost estimates to inform this parameter in the kidney model. The cost of standard transplantation was available for all three organs, but the cost of non-standard transplantation (i.e. EVMP transplantation) was only available for lungs. To estimate the cost of EVMP transplant for livers and kidneys therefore, we calculated the ratio of EVMP to standard lung transplantation costs and used this to estimate the cost of EVMP liver and kidney transplantation from standard transplantation costs. Average waiting list costs for livers and lungs were obtained directly from the literature. Average waiting list costs for kidneys were calculated as the weighted average of peritoneal and haemodialysis weighted by the proportion of individuals undergoing different dialysis interventions reported in the UK renal registry report.^17^ We excluded the cost of adrenaline given during cardiac arrest resuscitation to avoid double counting when combining results from the donor models with the trial-based cost-effectiveness results. This should not bias overall cost-effectiveness estimates as the cost of adrenaline is relatively insignificant in comparison to transplant related costs.

## Health utilities

Table A2 (Additional file 4) summarises health state utility values applied in the transplantation models. We extracted utility values from studies identified in our literature review and chosen based on similarity of the population generating the data to the UK population needing a transplant. Utility values in the lungs and liver models were based on UK data collected through the SF-36^5,6^ and the condition-specific liver disease quality of life questionnaire.^18^ Utility values in the kidney model came from three sources.^19-21^ The overall utility value for those on the kidney waiting list was calculated by summing utility values associated with different dialysis procedures weighted by the proportion of UK patients receiving each procedure.^17^ We assumed that patients waiting for kidney transplantation had already started dialysis and assigned a utility value from an Australian study of hospital haemodialysis and peritoneal dialysis^19^ and a Dutch study of a satellite and home-based haemodialysis population^20^. A health utility value following kidney transplantation was obtained from a Canadian study of screening for polyoma virus nephropathy^21^.

Table A2: Data inputs for transplant models

| **Parameter** | **Lung** | **Liver** | **Kidney** | **Distribution** | **Source** | **Population** |
| --- | --- | --- | --- | --- | --- | --- |
| *Organ supply (adrenaline v placebo)* |  |  |  |  |  |  |
| Randomised | 4015 v 3999 |  |  |  | PARAMEDIC2 trial data | UK |
| Transported to hospital | 2041 v 1227 |  |  |  |  |  |
| Survived to hospital discharge | 128 v 91 |  |  |  |  |  |
| Recipients of donor organ | 4 v 3 | 25 v 16 | 67 v 43 |  |  |  |
| Odds ratio for transplanting organ from cardiac arrest donor (95% CI) | 1.33 (0.30, 5.95) | 1.50 (0.80, 2.81) | 1.56 (1.06, 2.36) |  |  |  |
|  |  |  |  |  |  |  |
| *Probabilities* |  |  |  |  |  |  |
| Baseline DBD+DCD transplants per year | 196 | 995 | 2319 | Fixed | NHSBT Activity Report (Tables 7.11^a^, 8.8^b^,5.8^c^)^8^ | UK |
| Cardiac arrest contribution | 0.250 | 0.25 | 0.250 | Fixed | Nolan (2016)^11^ | UK |
| Proportion EVLP/EVMP | 0.10 | 0.10 | 0.20 | Beta | DEVELOP-UK,^5,6^ NICE TA165^16^ | UK, USA |
| *Waiting list transitions* |  |  |  |  |  |  |
| Number joining waiting list | 296 | 904 | 2873 | Fixed | NHSBT 2017/18 Cardiothoracic report (Figure 9.4)^9^  NHSBT 2017/18 Liver report (Figure 3.7)^10^  NHSBT 2017/18 main organ donor and transplant activity report (Figure 5.2)^8^ | UK |
| 1-year probability, transplant | 0.45 | 0.68 | 0.26 | Dirichlet |  |  |
| 1-year probability, death on list | 0.19 | 0.1 | 0.01 | Dirichlet |  |  |
| 1-year probability, removal from list | 0.01 | 0.04 | 0.01 | Dirichlet |  |  |
| 1-year probability, remaining on list | 0.35 | 0.18 | 0.72 | Dirichlet |  |  |
| 2-year probability, transplant | 0.54 | 0.77 | 0.42 | Dirichlet |  |  |
| 2-year probability, death on list | 0.25 | 0.12 | 0.03 | Dirichlet |  |  |
| 2-year probability, removal from list | 0.04 | 0.06 | 0.04 | Dirichlet |  |  |
| 2-year probability, remaining on list | 0.17 | 0.05 | 0.53 | Dirichlet |  |  |
| 3-year probability, transplant | 0.57 |  | 0.57 | Dirichlet |  |  |
| 3-year probability, death on list | 0.26 |  | 0.04 | Dirichlet |  |  |
| 3-year probability, removal from list | 0.08 |  | 0.06 | Dirichlet |  |  |
| 3-year probability, remaining on list | 0.09 |  | 0.33 | Dirichlet |  |  |
| 4-year probability, transplant |  |  | 0.65 | Dirichlet |  |  |
| 4-year probability, death on list |  |  | 0.06 | Dirichlet |  |  |
| 4-year probability, removal from list |  |  | 0.08 | Dirichlet |  |  |
| 4-year probability, remaining on list |  |  | 0.22 | Dirichlet |  |  |
| 5-year probability, transplant |  |  | 0.72 | Dirichlet |  |  |
| 5-year probability, death on list |  |  | 0.07 | Dirichlet |  |  |
| 5-year probability, removal from list |  |  | 0.10 | Dirichlet |  |  |
| 5-year probability, remaining on list |  |  | 0.11 | Dirichlet |  |  |
|  |  |  |  |  |  |  |
| *Post-transplantation survival* |  |  |  |  |  |  |
| Number at risk at day zero | 2805 |  |  | Fixed | NHSBT 2017/18 Cardiothoracic report (Table 13.1)^9^ | UK |
| 1-year overall survival | 0.778 |  |  | Beta | NHSBT 2017/18 Cardiothoracic report (Table 13.1)^9^ |  |
| 3-year overall survival | 0.700 |  |  |  | DEVELOP-UK^5,6^ |  |
| 5-year overall survival | 0.530 |  |  | Beta | NHSBT 2017/18 Cardiothoracic report (Table 13.1)^9^ |  |
| 10-year overall survival | 0.340 |  |  | Beta | 2017/18 Cardiothoracic (Table 13.1)^9^ |  |
| 19-year overall survival | 0.120 |  |  | Beta | DEVELOP-UK^5,6^ |  |
| 25-year overall survival | 0.000 |  |  | Beta | DEVELOP-UK UK^5,6^ |  |
| Median overall survival (95%CI) |  | 22 (19.6, 25.6) |  | Lognormal | Barber (2007)^12^ |  |
| Mean overall survival, years | 8.202 | 32 | 20.5 | Lognormal | DEVELOP-UK,^5,6^ Barber (2007)^12^ & Li (2016)^13^ |  |
|  |  |  |  |  |  |  |
| *Transplantation costs (£, 2017 prices)^1^* |  |  |  |  |  |  |
| Organ extraction & preservation | £0 | £17,832 | £17,832 | Gamma | DEVELOP-UK,^5,6^ Commission 2020 | UK |
| Waiting list costs per year | £23,104 | £24,520 | £34,292 | Gamma |  |  |
| Standard transplant | £50,203 | £58,396 | £43,138 | Gamma |  |  |
| EVLP/EVMP transplant | £133,342 | £155,335 | £114,747 | Gamma |  |  |
|  |  |  |  |  |  |  |
| *Post transplantation costs (£, 2017 prices)^1^* |  |  |  |  |  |  |
| Standard transplant, Year 1 | £9,405 | £13,375 | £17,691 | Gamma | DEVELOP-UK,^5,6^ Commission 2020 | UK |
| Standard transplant Year 2 | £3,696 | £5,573 | £6,764 | Gamma |  |  |
| Standard transplant, Year ≥3 | £3,400 | £5,573 | £6,764 | Gamma |  |  |
| EVMP, Year 1 | £5,739 | £13,375 | £17,691 | Gamma |  |  |
| EVMP, Year 2 | £2,255 | £5,573 | £6,764 | Gamma |  |  |
| EVMP, Year 3 | £2,075 | £5,573 | £6,764 | Gamma |  |  |
|  |  |  |  |  |  |  |
| *Utilities^1^* |  |  |  |  |  |  |
| Peritoneal dialysis (PD) |  |  | 0.569 | Beta | Howard (2010)^11^ | Australia |
| Hospital haemodialysis (HD) |  |  | 0.443 | Beta |  |  |
| Home haemodialysis |  |  | 0.810 | Beta | Gonzalez-Perez (2005)^20^ | Netherlands |
| Satellite haemodialysis |  |  | 0.810 | Beta |  |  |
| Waiting list | 0.563 | 0.659 | 0.559 | Beta | DEVELOP-UK,^4,5^ McLean (2017),^18^ Howard (2010)^19^ &  Gonzalez-Perez (2005)^20^ | UK, Australia, Netherlands |
| Post Standard transplant, Year 1 | 0.690 | 0.795 | 0.840 | Beta |  |  |
| Post Standard transplant, Year ≥2 | 0.728 | 0.730 | 0.840 | Beta |  |  |
| Post EVMP, Year 1 | 0.793 | 0.795 | 0.840 | Beta |  |  |
| Post EVMP, Year ≥2 | 0.782 | 0.730 | 0.840 | Beta |  |  |
|  |  |  |  |  |  |  |
| *Dialysis statistics* |  |  |  |  |  |  |
| Number on RRT at day90 |  |  | 6173 | Fixed | NEPHRON 2018 (Table 12a & Figure 1.9) | UK |
| Hospital HD at day90, proportion |  |  | 0.415 | Dirichlet |  |  |
| Home HD at day90, proportion |  |  | 0.040 | Dirichlet |  |  |
| Satellite HD at day90, proportion |  |  | 0.279 | Dirichlet |  |  |
| PD at day90, proportion |  |  | 0.205 | Dirichlet |  |  |
| Data sources (a=Lungs, b=Liver and c=Kidney)  EVLP/EVMP = Ex-vivo liver/machine perfusion,  ^1^Assumes uncertainty associated with cost and utilities equal 10% of the mean estimate when not reported | | | | | | |

## **Combining results across models**

The transplantation models described above simulate the impact of adrenaline use following cardiac arrest resuscitation compared with no adrenaline on supply of organs for transplantation. The desired outputs are the mean costs and benefits associated with the comparator treatments. From these, incremental cost-effectiveness ratios can be calculated to express the cost-effectiveness of adrenaline compared with no adrenaline for the recipient populations; however, these are not very meaningful in themselves, as they do not take into account impact of treatment on the cardiac arrest population, a large proportion of whom do not become organ donors. A more meaningful measure is one that integrates data across the populations of direct and indirect beneficiaries of adrenaline use in cardiac arrest resuscitation to generate a broader measure of costs and benefits to the health service and society. This requires an estimate of the size of the potential donor and recipient populations who stand to benefit directly and indirectly from adrenaline use in cardiac arrest resuscitation. Based on our base-case assumption that cardiac arrest contributes 25% of total deceased donor transplants (49 lungs, 249 livers and 580 kidneys) each year under current UK resuscitation practice and the reported number of transplantations in the 2017-18 activity year, we estimated that 81 adult lung, 309 liver and 731 kidney registrations would be eligible each year on average for cardiac arrest donated organs. The sheet labelled ‘Organ supply’ in the accompanying excel model workbook provides details of the calculations used to generate these estimates. To estimate the size of the potential donor population, we first calculated the probability that in hospital death from out-of-hospital cardiac arrest would generate successful transplantation. This is on the presumption that the acquisition of deceased out-of-hospital cardiac arrest donor organs only take place following death in hospital under current practice. We estimated this probability by dividing the 4 lung, 25 liver and 67 kidney transplantations by 1911 deaths in hospital among the 4015 patients randomised to the adrenaline arm of the PARAMEDIC2 trial. Next, we multiplied the probability of successful transplantation (0.0021 for lungs, 0.0131 for livers and 0.0351 for kidneys) by our estimate of cardiac arrest contribution to total deceased donor transplantations in the UK (49 lungs, 249 livers and 580 kidneys) to generate 23,410 cardiac arrest deaths for lungs, 19,014 for livers and 16,536 for kidneys. Since, the number of transplanted lungs, livers and kidneys come from the same pool of cardiac arrest deaths in hospital, we estimate the total in-hospitable out-of-hospital cardiac arrest deaths each year in the UK to lie between the three estimates. The average across the three estimates is 19,653 cardiac arrest deaths in hospital. To this we added 1974 and 130 representing the number of pre-hospital deaths and those who survived to hospital discharge in the adrenaline arm to generate a total predicted cardiac arrest population of 21,757 each year in the UK. We used the predicted population of direct (n=21,757) and indirect (81 lungs, 309 liver and kidney registrations) beneficiaries of adrenaline use in cardiac arrest resuscitation each year to scale up the cost-effectiveness estimates to a UK level before combining results across models.

## **Sensitivity analyses**

We conducted probabilistic sensitivity analysis to evaluate uncertainty in the data inputs on the cost-effectiveness of adrenaline. This involved generating 1000 random draws of model inputs from probability distributions to produce 1000 simulated replicates of the base-case results. From this, the probability that adrenaline is cost-effectiveness at threshold values ranging from £0 to £50,000 per QALY gained was calculated by formulating a net benefit function and counting the proportion of simulations generating a positive net benefit at the specified threshold.^3^ The distributions specified for each parameter are displayed in Table A1 and Table A2 (Additional file 4). The effect of adrenaline in cardiac arrest resuscitation on the supply of organs for transplantation was estimated using an odds ratio, which was assumed to follow an approximate normal distribution on the logarithmic scale. Probabilities and utility values were assigned Beta (or Dirichlet where there were more than two response categories) distributions to reflect scale of measurement for quantities constrained to lie in the interval 0-1.^3^ Gamma and or lognormal distributions were specified for parameters taking only positive values such as healthcare costs and length of survival following transplantation. Standard errors and confidence intervals were used to quantify the uncertainty surrounding input parameters where available. However, where not available, we assumed 10% of the mean as equivalent to lower and upper 95% confidence limits and calculated the standard errors assuming approximate normal distribution.

In addition to making the model probabilistic, one-way deterministic sensitivity analyses were conducted to explore the impact of modelling assumptions and data inputs on the cost-effectiveness of adrenaline. For the transplantation models, we varied the number of cardiac arrest donated grafts as a proportion of total deceased donor transplants from 25% in the base-case^11^ to values between 1% and 100%. This parameter controls the relative weighting used to pool results across the different populations that stand to benefit directly or indirectly from cardiac arrest resuscitation. It also controls the impact of adrenaline on pool of cardiac arrest donor organs available for transplantation and hence the costs and benefits that could be gained or forgone from adrenaline use in cardiac arrest resuscitation. Increasing the proportion of grafts from cardiac arrest donors favours adrenaline whereas decreasing it would favour placebo. A recent Dutch study suggested that the cardiac arrest patients donated almost 10% of 6322 deceased kidney grafts between 2007 and 2017.^22^ This represented 4.5% of grafts donated after brain death (DBD) and 17.3% donated after circulatory death (DCD). An analysis of UKBT transplant activity between October 2009 and December 2010 suggested cardiac arrest as cause of death in 1.5% of DCD transplants but this could potentially rise to 6.5%.^23^ The proportion of transplants requiring in situ or EVMP/EVLP reconditioning prior to transplantation of 10% for lungs and livers and 45% for kidneys were doubled and halved in separate analyses. This parameter controls the cost of transplantation due to the high cost of grafting reconditioned organs compared to standard transplantation. Increasing this parameter is therefore less favourable to adrenaline as it increases costs in the adrenaline arm from greater transplant activity compared with placebo. Sensitivity analysis explored the impact of halving and doubling proportion of transplants that require high cost reconditioning procedures. The maximum time allowed on the waiting list was set to 10 years after which patients automatically drop out and no longer accrue costs and benefits. This favours adrenaline because of the predicted reduction in organ supply from withdrawing adrenaline use in cardiac arrest resuscitation. Sensitivity analysis explored the impact of assuming that patients receive automatic transplant after 10 years on the waiting list instead of being removed from the waiting list. Analyses were also conducted that halved and doubled health state utility values.

For the long-term extrapolation modelling, the primary source of uncertainty remained the annual probability of death for those surviving beyond the first year after the cardiac arrest event. In the base-case analysis, this was estimated using the probability of death during the 9-12 month period, given survival to 9 months after the cardiac arrest event or age and sex-adjusted UK mortality statistics, whichever was greatest stratified.

# Results

## Base-case analysis results

Table 3 and Table 4 presents base-case results for the cardiac arrest population and potential recipients of cardiac arrest donated organs. For cardiac arrest resuscitation, the trial-based analysis suggested that adrenaline was unlikely to be cost-effective with an incremental cost-effectiveness ratio (ICER) close to £1,693,003 per QALY gained compared with placebo over a six-month time horizon. Extrapolating the within-trial analysis beyond the 6-month trial follow-up reduced the ICER to £644,308 over a one-year time horizon and to £81,070 per QALY gained over the lifetime of cardiac arrest survivors (Table 3).

Results are also presented for recipients of transplant organs donated by cardiac arrest patients (Table A3, Additional file 4) however, as explained in the methods, these are not meaningful in themselves and so we have generated cost per QALY estimates for the combined population of cardiac arrest and transplant recipients. Based on the number of transplanted livers, lungs and kidneys donated by PARAMEDIC2 trial participants and the steady state assumption, our modelling predicts that, on average, 21,757 individuals would be eligible for adrenaline following cardiac arrest resuscitation to produce 25% of liver, lung and kidney transplantations each year. We also predicted 1,122 individuals joining the organ transplant list each year would be eligible for one of the donated organs of which 81 (7.2%) would be eligible for lung, 309 (27.6%) for liver and 731 (65.2%) for kidney transplantation. These numbers were used scale up results to a UK population level and generate estimates of cost-effectiveness for the combined population of recipients and donors. The combined result suggests that adrenaline was associated with incremental costs of £49,759,481 and incremental QALYs of 3093 discounted at 3.5% per annum on average over a lifetime time horizon. The incremental cost-effectiveness ratio for the combined result was £16,086 per QALY gained for adrenaline compared with placebo (Table A3, Additional file 4). The probabilistic analysis located the majority of the 1000 simulated ICERs in the northeast quadrant of the cost-effectiveness plane (Figure 1), suggesting adrenaline was more costly and more effective than placebo, on average. The average combined ICER in the probabilistic analysis was £16,899 per QALY gained for adrenaline compared with placebo. The probability that adrenaline was cost-effective compared with placebo was 41% at a £15,000 per QALY cost-effectiveness threshold, 68% at a £20,000 per QALY cost-effectiveness threshold and 90% at a £30,000 per QALY cost-effectiveness threshold (Table A3 and Figure 1, plot C ).

Table A3: Base-case results combining direct economic effects over the lifetimes of survivors and indirect economic effects in organ recipients presented at UK population level

|  | **Adrenaline** | | **Placebo** | | **Cost-effectiveness** | | | **Probability adrenaline is cost-effective at** | | |
| --- | --- | --- | --- | --- | --- | --- | --- | --- | --- | --- |
| Analysis^1^ | Mean costs | Mean QALYs | Mean costs | Mean QALYs | Incremental costs | Incremental QALYs | ICER | £15,000 / QALY | £20,000 / QALY | £30,000 /QALY |
| Trial-based CUA (*n=21,757*)^2^ | £78,130,649 | 54 | £48,662,440 | 37 | £29,468,209 | 17 |  |  |  |  |
| Extrapolation beyond trial follow-up (*n=21,757*)^2^ | £115,497,429 | 2414 | £76,885,801 | 1938 | £38,611,628 | 476 |  |  |  |  |
| Lung transplant (*n=81*)^2^ | £5,306,026 | 228 | £5,175,751 | 201 | £130,275 | 28 |  |  |  |  |
| Liver transplant (*n=309*)^2^ | £47,550,753 | 3038 | £39,979,944 | 2250 | £7,570,808 | 788 |  |  |  |  |
| Kidney transplant (*n=731*)^2^ | £147,284,791 | 7262 | £143,838,021 | 5461 | £3,446,770 | 1801 |  |  |  |  |
|  |  |  |  |  |  |  |  |  |  |  |
| Combined cost-effectiveness |  |  |  |  |  |  |  |  |  |  |
| Trial-based analysis (*n=21,757*)^2^ | £78,130,649 | 54 | £48,662,440 | 37 | £29,468,209 | 17 | £1,693,003 | 0.00 | 0.00 | 0.00 |
| Trial-based + extrapolation ((*n=21,757*)^2^ | £115,497,429 | 2414 | £76,885,801 | 1938 | £38,611,628 | 476 | £81,070 | 0.04 | 0.05 | 0.10 |
| Trial-based + extrapolation + lung (n=81) | £120,803,455 | 2642 | £82,061,552 | 2138 | £38,741,903 | 504 | £76,859 | 0.03 | 0.05 | 0.10 |
| Trial-based + extrapolation + liver (n=309) | £163,048,181 | 5452 | £116,865,746 | 4187 | £46,182,436 | 1264 | £36,533 | 0.06 | 0.13 | 0.35 |
| Trial-based + extrapolation + kidney (n=731) | £262,782,220 | 9676 | £220,723,823 | 7398 | £42,058,397 | 2278 | £18,466 | 0.34 | 0.57 | 0.82 |
| Trial-based + extrapolation + lung + liver + kidney | £315,638,999 | 12942 | £265,879,518 | 9849 | £49,759,481 | 3093 | £16,086 | 0.41 | 0.68 | 0.90 |
| ^1^The time horizon is life-time except for the within-trial analysis where it is 6-months  ^2^n refers to the eligible patient population per year predicted from the modelling; for transplant models, refers to predicted numbers joining waiting list each year | | | | | | | | | | |

## Results of sensitivity analyses

Table A4 (Additional file 4) display the deterministic sensitivity analyses results for the model extrapolating within-trial cost-effectiveness to lifetime of cardiac arrest survivors. Varying assumptions and data inputs used to extrapolate cost-effectiveness beyond the PARAMEDIC2-trial follow-up to a lifetime horizon for patients who survived cardiac arrest did not significantly change the combined ICER value.

Table A5 (Additional file 4) display the deterministic sensitivity analyses results for the combined cost-effectiveness analysis. The results suggest the combined base-case ICER is only sensitive to the odds ratio that estimated the impact of adrenaline compared with placebo on the supply of organs for transplantation. Using the upper 95% confidence limit value had minimal impact on the base-case ICER but using the lower 95% confidence limits (odds ratio of 0.29 for lungs, 0.8 for livers and 1.06 for kidneys) increased the ICER from £16,086 to £183,251 per QALY gained for adrenaline compared with placebo (Table A5, Additional file 4). In all other tested scenarios, the ICER remained below £40,000 per QALY gained.

In the base-case analysis, the probability of receiving an organ donated by a cardiac arrest patient was set to 25% based on analysis of ICNARC data.^11^ Changing this to 10% and 1.5% based on data reported elsewhere^22,23^ changed the combined base-case ICER from £16,086 to £17,506 and £28,378 per QALY gained, respectively. Assuming the 25% cardiac arrest contribution affects only DCD transplants also had minimal impact with the ICER changing to £16,129 per QALY gained. Figure A1 displays the impact of varying the contribution of cardiac arrest donors to total transplants each year from 0% to 100% on the combined ICER. It suggests an exponential decay relationship with the ICER decreasing sharply to a plateau at about the £17,506 per QALY mark, which corresponds to approximately 10% of deceased donor transplantations coming from cardiac arrest patients.

Figure A1: Impact of varying the proportion of transplanted livers, lungs and kidneys from deceased donor where cardiac arrest is cause of death

In the base-case analysis, the proportion of organs requiring ex-vivo machine perfusion (EVMP) in the case of kidneys and livers and ex-vivo lung perfusion (EVLP) reconditioning prior to transplantation was set to 10% for lungs and livers and 20% for kidneys. This parameter controls the cost of transplantation so that increasing it favours placebo as a larger proportion of transplanted organs undergo high cost reconditioning prior to surgery. Sensitivity analysis doubling the rate of EVMP/EVLP transplants to 20% for lungs and livers and 40% for kidneys increased the ICER from £16,086 to £17,277 per QALY gained, whist halving the rate of EVMP transplants decreased the ICER to £15,491 per QALY gained.

In the base-case analysis, the maximum waiting time for an organ was set at 10 years beyond which patients drop out and no longer accrue costs and benefits. Overall, the proportion of patients still waiting for transplant 10 years from joining the list was small under base-case assumptions – 0% for livers, 0.11% for lungs and 0.34% for kidneys in the adrenaline arm and 3% for livers, 2.33% for lungs and 10% for kidneys in the placebo arm. Sensitivity analysis setting the probability of transplantation at 10 years on the waiting list to 1.00 decreased the base-case ICER to £17,008 per QALY gained.

Table S10: Impact of alternative modelling assumptions and data inputs used to extrapolate beyond PARAMEDIC2 trial follow-up

|  | **Adrenaline** | | **Placebo** | | **Cost-effectiveness** | | |
| --- | --- | --- | --- | --- | --- | --- | --- |
| Analysis | Mean costs | Mean QALY | Mean costs | Mean QALY | Incremental Costs | Incremental QALY | ICER |
| Base-case | £315,637,135 | 12942 | £265,878,278 | 9849 | £49,758,858 | 3093 | £16,086 |
| Changed 1-year survival with good function in adrenaline arm from 2.3% to 2.1% and placebo arm from 1.9% to 1.6% | £313,705,327 | 12769 | £266,432,157 | 9898 | £47,273,171 | 2871 | £16,465 |
| Doubled 1-year survival with poor function in adrenaline arm to 1% and placebo arm to 0.6% | £324,626,260 | 12978 | £271,575,510 | 9872 | £53,050,750 | 3106 | £17,077 |
| Annual treatment costs, survival with good function changed from £7,907 to £5,282, poor function from £24,457 to £16,141 (lower 95% confidence limit) | £304,638,825 | 12942 | £257,482,490 | 9849 | £47,156,335 | 3093 | £15,245 |
| Annual treatment costs, survival with good function changed from £7,907 to £11,084, poor function from £24,457 to £34,641 (upper 95% confidence limit) | £328,257,422 | 12942 | £275,496,884 | 9849 | £52,760,537 | 3093 | £17,057 |
| Change utility associated with good function from 0.707 to 0.671, poor function from 0.0982 to -0.0037 (lower 95% confidence limit) | £315,637,135 | 12789 | £265,878,278 | 9731 | £49,758,858 | 3059 | £16,269 |
| Change utility associated with good function from 0.707 to 0.741, poor function from 0.0982 to 0.1964 (upper 95% confidence limit) | £315,637,135 | 13091 | £265,878,278 | 9964 | £49,758,858 | 3128 | £15,910 |

Table A5: Impact of alternative modelling assumptions and data inputs used to inform the transplantation models

|  | **Adrenaline** | | **Placebo** | | **Cost-effectiveness** | | |
| --- | --- | --- | --- | --- | --- | --- | --- |
| Analysis | Mean costs | Mean QALYs | Mean costs | Mean QALYs | Incremental Costs | Incremental QALYs | ICER |
| Base case combined ICER | £315,638,999 | 12942 | £265,879,518 | 9849 | £49,759,481 | 3093 | £16,086 |
| Changed % transplants, cardiac arrest donor from 0.25 to 0.1 (Schaapherder 2018)^35^ | £132,956,965 | 5317 | £110,812,857 | 4052 | £22,144,107 | 1265 | £17,506 |
| Changed % transplants, cardiac arrest donor from 0.25 to 0.015 (Manara 2010)^36^ | £29,437,145 | 996 | £22,941,750 | 767 | £6,495,396 | 229 | £28,378 |
| Applied 25% cardiac arrest contribution to DCD transplants only | £107,383,351 | 4361 | £91,979,392 | 3318 | £15,403,960 | 1043 | £14,765 |
| Patients receive transplant after 10-years on waiting list | £315,691,926 | 12947 | £277,037,800 | 10674 | £38,654,126 | 2273 | £17,008 |
| Automatic transplant after year2 (liver), year3(lung) and year5 (kidney) | £318,753,080 | 13259 | £287,385,893 | 12188 | £31,367,187 | 1070 | £29,305 |
| Automatic removal from waiting list after year2 (liver), year3 (lung) and year5 (kidney) | £303,232,800 | 12254 | £238,931,091 | 9045 | £64,301,709 | 3209 | £20,039 |
| Halved % EVMNP transplants | £310,353,662 | 12941 | £262,439,646 | 9848 | £47,914,016 | 3093 | £15,491 |
| Doubled % EVMP transplants to 100% | £326,209,673 | 12944 | £272,759,263 | 9850 | £53,450,410 | 3094 | £17,277 |
| Halved all costs | £211,474,796 | 12942 | £168,732,798 | 9849 | £42,741,999 | 3093 | £13,818 |
| Doubled all costs | £548,527,909 | 12942 | £476,072,130 | 9849 | £72,455,779 | 3093 | £23,424 |
| Halved all utilities | £315,638,999 | 7678 | £265,879,518 | 5893 | £49,759,481 | 1785 | £27,880 |
| Halved mean post-transplantation survival | £285,829,574 | 9152 | £246,512,417 | 7381 | £39,317,157 | 1770 | £22,213 |
| Doubled mean post-transplantation survival | £348,626,147 | 17144 | £287,292,996 | 12583 | £61,333,152 | 4561 | £13,448 |
| Changed treatment effect odds ratio to lower 95% CI | £353,966,228 | 13910 | £307,488,444 | 13656 | £46,477,784 | 254 | £183,251 |
| Changed treatment effect odds ratio to upper 95% CI | £315,638,999 | 12942 | £256,364,491 | 7953 | £59,274,508 | 4989 | £11,881 |

## **References**

1. R Core Team. R: A language and environment for statistical computing. R Foundation for Statistical Computing, Vienna, Austria; 2019. URL https://www.R-project.org/.
2. Filipovic-Pierucci A, Zarca K, Durand-Zaleski I. Markov Models For Health Economic Evaluation Modelling In R With The Heemod Package. *Value in Health* 2016;19(7):A369. doi: 10.1016/j.jval.2016.09.133
3. Briggs AC, Karl. Sculpher, Mark. Decision Modelling for Health Economic Evaluation. Oxford: Oxford University Press 2006.
4. Andersen LW, Holmberg MJ, Granfeldt A, et al. Cost-effectiveness of public automated external defibrillators. *Resuscitation* 2019;138:250-58. doi: 10.1016/j.resuscitation.2019.03.029
5. Fisher A, Andreasson A, Chrysos A, et al. An observational study of Donor Ex Vivo Lung Perfusion in UK lung transplantation: DEVELOP-UK. *Health technology assessment* 2016;20(85) doi: 10.3310/hta20850
6. McMeekin N, Chrysos AE, Vale L, et al. Incorporating ex-vivo lung perfusion into the UK adult lung transplant service: an economic evaluation and decision analytic model. *BMC Health Services Research* 2019;19(1):326. doi: 10.1186/s12913-019-4154-6
7. National Istitute for Health and Care Excellence. Guide to the methods of technology appraisal 2013. Avaialable from <http://publications.nice.org.uk/pmg9>, 2013.
8. NHS Blood and Transplant. Organ Donation and Transplantation Activity Report 2017/18. Available from [https://nhsbtdbe.blob.core.windows.net/umbraco-assets/1848/transplant-activity-report-2017-2018.pdf. Accessedd 13/08/2019. 2018](https://nhsbtdbe.blob.core.windows.net/umbraco-assets/1848/transplant-activity-report-2017-2018.pdf.%20Accessedd%2013/08/2019.%202018)
9. NHS Blood and Transplant. Annual report on cardiothoracic organ transplantation report for 2017/2018. Available from <https://nhsbtdbe.blob.core.windows.net/umbraco-assets-corp/12252/nhsbt-cardiothoracic-transplantation-annual-report-2017-2018.pdf>, 2018.
10. NHS Blood and Transplant. Annual report on liver transplantation report for 2017/2018. Available from <https://nhsbtdbe.blob.core.windows.net/umbraco-assets-corp/12250/nhsbt-liver-transplantation-annual-report-2017-2018.pdf>. Accessed 13/08/2019, 2018.
11. Nolan JP, Ferrando P, Soar J, et al. Increasing survival after admission to UK critical care units following cardiopulmonary resuscitation. *Critical Care* 2016;20(1):219. doi: 10.1186/s13054-016-1390-6
12. Barber K, Blackwell J, Collett D, et al. Life expectancy of adult liver allograft recipients in the UK. 2007;56(2):279-82. doi: 10.1136/gut.2006.093195 %J Gut
13. Li B, Cairns JA, Robb ML, et al. Predicting patient survival after deceased donor kidney transplantation using flexible parametric modelling. *BMC Nephrology* 2016;17(1):51. doi: 10.1186/s12882-016-0264-0
14. Curtis L. Unit Costs of Health and Social Care 2017, Personal Social Services Research Unit, University of Kent, Canterbury. Available from <http://www.pssru.ac.uk/project-pages/unit-costs/2017/>. 2017
15. NHS Scotland. Commissioning Transplantation to 2020. Subgroup of the National Specialist Services Committee Final Report. Available from <https://www.nsd.scot.nhs.uk/publications/Servicereviews/CTT2020%20final%20report.pdf>. Accessed 13/08/2019, 2014.
16. National Institute for Health and Care Excellence (NICE) (2018) Final appraisal determination: Machine perfusion systems and cold static storage of kidneys from deceased donors. Available online from: [https://www.nice.org.uk/guidance/ta165/documents/organ-preservation-renal-machine-perfusion-static-storage-final-appraisal-determintation2. Accessed 03/02/2020](https://www.nice.org.uk/guidance/ta165/documents/organ-preservation-renal-machine-perfusion-static-storage-final-appraisal-determintation2.%20Accessed%2003/02/2020).
17. Byrne C CF, Castledine C, Davenport A, Dawnay A, Fraser S, Maxwell H, Medcalf JF, Wilkie M,, AJ W. NEPHRON 2018;139 (suppl1) UK Renal Registry 20th Annual Report of the Renal Association. Available from <https://www.renalreg.org/wp-content/uploads/2018/06/20th-Annual-Report_web_book.pdf>. Accessed 20/08/2019. 2018
18. McLean KA, Camilleri-Brennan J, Knight SR, et al. Decision modeling in donation after circulatory death liver transplantation. 2017;23(5):594-603. doi: 10.1002/lt.24715
19. Howard K, White S, Salkeld G, et al. Cost-Effectiveness of Screening and Optimal Management for Diabetes, Hypertension, and Chronic Kidney Disease: A Modeled Analysis. *Value in Health* 2010;13(2):196-208. doi: 10.1111/j.1524-4733.2009.00668.x
20. Gonzalez-Perez JG, Vale L, Stearns SC, et al. Hemodialysis for end-stage renal disease: A cost-effectiveness analysis of treatment options. *International Journal of Technology Assessment in Health Care* 2005;21(1):32-39. doi: 10.1017/S026646230505004X [published Online First: 2005/03/02]
21. Kiberd BA. Screening to Prevent Polyoma Virus Nephropathy: A Medical Decision Analysis. 2005;5(10):2410-16. doi: 10.1111/j.1600-6143.2005.01034.x
22. Schaapherder A, Wijermars LGM, de Vries DK, et al. Equivalent Long-term Transplantation Outcomes for Kidneys Donated After Brain Death and Cardiac Death: Conclusions From a Nationwide Evaluation. *EClinicalMedicine* 2018;4-5:25-31. doi: <https://doi.org/10.1016/j.eclinm.2018.09.007>
23. Manara AR, Murphy PG, O'Callaghan G. Donation after circulatory death. *BJA: British Journal of Anaesthesia* 2012;108(suppl_1):i108-i21. doi: 10.1093/bja/aer357 %J BJA: British Journal of Anaesthesia

1. An alternative approach that would yield exactly the same result is to apply cardiac arrest contribution under current practice of 25% to the number of transplantations (179 lungs, 727 livers and 2277 deceased donor kidneys) predicted to take place over 10-years in the section above. [↑](#footnote-ref-1)
2. Strictly this should be a rate ratio (as number of successful transplants per donor can be greater than 1). However as only a small proportion of cardiac arrest patients go on to donate, it’s reasonable to assume 0/1 donated organ cardiac arrest patient and from this calculate odds ratio as a conservative estimate of the treatment effect (i.e. transplant rate ratio for adrenaline compared with placebo). [↑](#footnote-ref-2)
