# Supplementary material for: Cost-effectiveness of adrenaline for out-of-hospital cardiac arrest
Source: Crit Care. 2020 Sep 27;24:579. doi: 10.1186/s13054-020-03271-0 (PMC7520962; doi:10.1186/s13054-020-03271-0)
Supplement: Supplementary file 6 — Additional file 6. Resources use for NHS and personal and social services by trial arm in the 6 month post-randomisation period; all patients. Resource use results table, within-trial economic evaluation. [file 13054_2020_3271_MOESM6_ESM.zip › 2020-06-27-Additional file 6.docx]

Additional 6: Resources use for NHS and personal and social services by trial arm in the 6 month post-randomisation period; all patients

|  | **Adrenaline (n=4,015)** | | **Placebo (n=3,999)** | | **Adrenaline versus Placebo** | |
| --- | --- | --- | --- | --- | --- | --- |
| Category | Participants with complete data (n) | Mean (SE) per 1000 individuals | Participants with complete data (n) | Mean (SE) per 1000 individuals | Mean difference (bootstrap 95% CI) per 1000 individuals | P-value |
| *Emergency response (minutes)* |  |  |  |  |  |  |
| Time 1 (arrival at scene to departure) | 4013 | 60279 (779) | 3999 | 58745 (547) | 1534.3 (-199, 3392) | 0.1 |
| Time 2^1^ (departure from scene to arrival at hospital or mortuary) | 4011 | 70457 (234) | 3992 | 70039 (297) | 418 (-332, 1152) | 0.244 |
|  |  |  |  |  |  |  |
| *Inpatient stay – initial admission (days)* |  |  |  |  |  |  |
| Emergency department | 4006 | 510 (9) | 3996 | 309 (8) | 201 (179, 224) | <0.001 |
| General ward | 4013 | 792 (98) | 3998 | 496 (72) | 296 (78, 536) | 0.008 |
| Intensive care unit | 4013 | 910 (59) | 3999 | 472 (47) | 438 (298, 592) | <0.001 |
|  |  |  |  |  |  |  |
| *Inpatient stay – repeat admissions (days)* |  |  |  |  |  |  |
| Emergency department | 3958 | 8 (2) | 3952 | 6 (2) | 2 (-4, 7) | 0.676 |
| General ward | 3971 | 90 (27) | 3966 | 102 (52) | -12 (-147, 85) | 0.91 |
| Intensive care unit | 4006 | 8 (8) | 3989 | 0 (0) | 8 (0, 25) | 0.276 |
|  |  |  |  |  |  |  |
| *Outpatient attendance (visits)* |  |  |  |  |  |  |
| Cardiology | 3956 | 32 (5) | 3950 | 23 (5) | 10 (-5, 23) | 0.214 |
| Cardiac rehab | 3958 | 63 (16) | 3951 | 56 (16) | 7 (-35, 48) | 0.816 |
| Nursing/residential home | 3957 | 142 (71) | 3951 | 45 (33) | 96 (-43, 271) | 0.178 |
| Other outpatient attendance | 3957 | 30 (9) | 3950 | 37 (18) | -7 (-47, 30) | 0.838 |
|  |  |  |  |  |  |  |
| *Primary health care contact (number of contacts)* |  |  |  |  |  |  |
| District nurse | 3959 | 80 (41) | 3953 | 13 (5) | 66 (4, 158) | 0.024 |
| GP, surgery visit | 3959 | 57 (10) | 3953 | 39 (7) | 18 (-6, 42) | 0.14 |
| GP, home visit | 3959 | 7 (3) | 3953 | 4 (2) | 4 (-3, 10) | 0.35 |
| GP, telephone consultation | 3958 | 3 (1) | 3951 | 4 (1) | -1 (-4, 3) | 0.616 |
| Practice Nurse | 3958 | 1 (1) | 3951 | 0 (0) | 1 (0, 2) | 0.596 |
| Physiotherapy | 3958 | 38 (36) | 3951 | 0 (0) | 38 (0, 113) | 0.084 |
| Occupational Therapy | 3958 | 1 (1) | 3951 | 1 (1) | 0 (-2, 1) | 0.392 |
| Social worker | 3959 | 7 (2) | 3953 | 4 (2) | 3 (-3, 9) | 0.458 |
| Speech therapy | 3959 | 6 (3) | 3953 | 11 (8) | -5 (-24, 8) | 0.562 |
| Psychiatrist | 3959 | 1 (1) | 3953 | 1 (1) | 0 (-3, 2) | 0.712 |
| Psychology | 3959 | 3 (1) | 3953 | 7 (4) | -4 (-15, 3) | 0.27 |
| Counsellor | 3959 | 3 (3) | 3953 | 1 (1) | 3 (-1, 9) | 0.574 |
| Home care worker | 3959 | 105 (55) | 3953 | 18 (14) | 87 (-10, 214) | 0.096 |
| Lunch or social club | 3959 | 4 (3) | 3953 | 7 (4) | -2 (-13, 7) | 0.636 |
| Self-help groups | 3959 | 50 (33) | 3953 | 9 (7) | 41 (-12, 116) | 0.22 |
| Meals and laundry | 3959 | 93 (49) | 3953 | 27 (16) | 65 (-22, 180) | 0.182 |
| Other community care | 3958 | 7 (6) | 3951 | 0 (0) | 7 (0, 24) | 0.138 |
|  |  |  |  |  |  |  |
| *Medications (item)* |  |  |  |  |  |  |
| Medication, number of items | 3956 | 4155 (24) | 3951 | 4096 (16) | 59 (4, 113) | 0.042 |
|  |  |  |  |  |  |  |
| *Aids and adaptations (per item/pair where appropriate)* |  |  |  |  |  |  |
| Hoist | 3958 | 1 (1) | 3952 | 0 (0) | 1 (0, 3) | 0.448 |
| Wheelchair | 3958 | 2 (1) | 3952 | 1 (1) | 1 (-1, 3) | 0.426 |
| Walking aid | 3958 | 3 (1) | 3952 | 4 (1) | -1 (-4, 2) | 0.338 |
| Hand aid | 3958 | 0 (0) | 3952 | 1 (1) | -1 (-2, 0) | 0.042 |
| Stair rail | 3958 | 0 (0) | 3952 | 0 (0) | 0 (-1, 0) | <0.001 |
| Other aids and adaptations (ICD with telemtry (remote monitoring), ICD, Heart monitor, PEG feed pump, Remote cardiac monitor) | 3958 | 9 (2) | 3952 | 3 (1) | 5 (0, 11) | 0.054 |
| ^1^We added 60 minutes (step down or restock time) to account for time that ambulance crew use to restock before they are available for the next assignment. This is based on asking ambulance crew during one of the meetings to discuss the trial results | | | | | | |
