# Supplementary material for: Cost-effectiveness of adrenaline for out-of-hospital cardiac arrest
Source: Crit Care. 2020 Sep 27;24:579. doi: 10.1186/s13054-020-03271-0 (PMC7520962; doi:10.1186/s13054-020-03271-0)
Supplement: Supplementary file 6 — Additional file 6. Resources use for NHS and personal and social services by trial arm in the 6 month post-randomisation period; all patients. Resource use results table, within-trial economic evaluation. [file 13054_2020_3271_MOESM6_ESM.zip › Additional file 6.docx]

Additional file 6: Cost-effectiveness (£, 2017 prices) of adrenaline based on within-trial economic evaluation; sensitivity analyses and subgroup analyses results

**Sensitivity analyses results (£, 2017 prices)**

|  | **Adrenaline** | | **Placebo** | | **Cost-effectiveness** | | | **Probability adrenaline is cost-effective at** | | |
| --- | --- | --- | --- | --- | --- | --- | --- | --- | --- | --- |
| Analysis model | Mean costs (£), SE | Mean QALYs, SE | Mean costs (£), SE | Mean QALYs, SE | **Incremental costs (95% CI)** | **Incremental QALYs (95% CI)** | **ICER** | **pCE, £15,000/ QALY** | **pCE, £20,000/ QALY** | **pCE, £30,000/ QALY** |
| Unadjusted multiple imputation | 4566 (166) | 0.0076 (0.0008) | 3229 (166) | 0.0067 (0.0008) | 1337 (877, 1797) | 0.001 (-0.0012, 0.0031) | 1401872 | 0 | 0 | 0 |
| Adjusted complete case | 3082 (369) | -0.0007 (0.002) | 2067 (371) | -0.0014 (0.002) | 1015 (725, 1306) | 0.0007 (-0.0008, 0.0023) | 1377745 | 0 | 0 | 0 |
| Unadjusted complete case | 3943 (106) | 0.0064 (0.0006) | 2905 (106) | 0.0057 (0.0006) | 1038 (745, 1332) | 0.0007 (-0.0009, 0.0022) | 1521586 | 0 | 0 | 0 |
| Parameter estimates via linear regression implemented within a within a bootstrap | 3592 (522) | 0.0024 (0.0025) | 2292 (527) | 0.0017 (0.0025) | 1300 (846, 1755) | 0.0007 (-0.0014, 0.0029) | 1784993 | 0 | 0 | 0 |
| QALYS based on MRS data 6 months | 3591 (541) | 0.0031 (0.0026) | 2285 (545) | 0.0018 (0.0026) | 1306 (836, 1776) | 0.0012 (-0.001, 0.0035) | 1069119 | 0 | 0 | 0 |
| QALYs derived assuming baseline utility of -0.042 (unconscious state) | 3592 (530) | 0.0018 (0.0024) | 2292 (535) | 0.0015 (0.0024) | 1300 (839, 1761) | 0.0003 (-0.0018, 0.0024) | 4218224 | 0 | 0 | 0 |
| Societal costs, 6 months | 3638 (541) | 0.0025 (0.0025) | 2228 (545) | 0.0017 (0.0026) | 1410 (940, 1880) | 0.0008 (-0.0014, 0.003) | 1828271 | 0 | 0 | 0 |
| QALYs based on EQ-5D/mRS, 12 months NHS/PSS costs | 3741 (536) | 0.006 (0.0049) | 2330 (541) | 0.0038 (0.005) | 1411 (946, 1876) | 0.0022 (-0.0021, 0.0065) | 644308 |  |  |  |
| QALYS based on MRS data, 12 months NHS/PSS costs | 3742 (538) | 0.0068 (0.005) | 2327 (542) | 0.0041 (0.0051) | 1415 (948, 1881) | 0.0027 (-0.0017, 0.007) | 528477 | 0 | 0 | 0 |
| QALYs based on EQ-5D/mRS, 12 months societal costs | 3768 (571) | 0.0059 (0.005) | 2259 (576) | 0.0037 (0.005) | 1509 (1014, 2004) | 0.0022 (-0.0021, 0.0066) | 679317 | 0 | 0 | 0 |
| Excluded stand down and restock time in emergency response cost calculations | 2989 (526) | 0.002 (0.0025) | 1684 (530) | 0.0012 (0.0025) | 1305 (844, 1766) | 0.0007 (-0.0015, 0.0029) | 1835618 | 0 | 0 | 0 |
| Excluded estimated cost of transporting deceased patients to nearest hospital mortuary if patient died at scene of cardiac arrest and in a public place | 3445 (525) | 0.0019 (0.0025) | 2146 (529) | 0.0012 (0.0025) | 1299 (838, 1760) | 0.0007 (-0.0014, 0.0029) | 1794795 | 0 | 0 | 0 |
| Excluded stand down/restock time and cost of transporting patients to nearest mortuary in emergency response cost calculations | 2989 (526) | 0.002 (0.0025) | 1684 (530) | 0.0012 (0.0025) | 1305 (844, 1766) | 0.0007 (-0.0015, 0.0029) | 1835618 | 0 | 0 | 0 |

Cost-effectiveness (£, 2017 prices) of adrenaline based on within-trial economic evaluation; sub-group analyses

|  | **Adrenaline** | | **Placebo** | | **Cost-effectiveness** | | | **Probability adrenaline is cost-effective at** | | |
| --- | --- | --- | --- | --- | --- | --- | --- | --- | --- | --- |
|  | **Mean costs (£), SE** | **Mean QALYs, SE** | **Mean costs (£), SE** | **Mean QALYs, SE** | **Incremental costs (95% CI)** | **Incremental QALYs (95% CI)** | **ICER** | **pCE, £15,000/ QALY** | **pCE, £20,000/ QALY** | **pCE, £30,000/ QALY** |
| ***Cause of cardiac arrest*** |  |  |  |  |  |  |  |  |  |  |
| Non-medical | 3491 (660) | 0.0014 (0.0031) | 2403 (702) | 0.0029 (0.0033) | 1088 (-563, 2740) | -0.0015 (-0.0092, 0.0062) | Dominated | 0.085 | 0.079 | 0.079 |
| Medical | 3763 (383) | 0.005 (0.0018) | 2438 (702) | 0.004 (0.0033) | 1325 (-1062, 3713) | 0.001 (-0.0102, 0.0122) | 1356042 | 0.141 | 0.14 | 0.147 |
| ***Age*** |  |  |  |  |  |  |  |  |  |  |
| ≤ 60 years | 5550 (552) | 0.0087 (0.0026) | 3234 (561) | 0.0059 (0.0026) | 2316 (1614, 3018) | 0.0028 (-0.0005, 0.0061) | 823606 | 0 | 0 | 0 |
| > 60 years | 2729 (570) | 0.0001 (0.0027) | 2272 (561) | 0.0011 (0.0026) | 457 (-722, 1636) | -0.001 (-0.0065, 0.0046) | Dominated | 0.199 | 0.195 | 0.195 |
| ***Gender*** |  |  |  |  |  |  |  |  |  |  |
| Female | 3434 (563) | 0.0022 (0.0026) | 2432 (565) | 0.002 (0.0026) | 1002 (213, 1790) | 0.0002 (-0.0034, 0.0039) | 4145700 | 0.007 | 0.007 | 0.004 |
| Male | 3690 (521) | 0.0035 (0.0024) | 2220 (565) | 0.0024 (0.0026) | 1470 (213, 2728) | 0.0011 (-0.0048, 0.0069) | 1389015 | 0.015 | 0.015 | 0.016 |
| ***EMS arrival at scene to administration of first dose*** |  |  |  |  |  |  |  |  |  |  |
| ≤ 10 minutes | 3399 (600) | 0.0031 (0.0028) | 2478 (605) | 0.001 (0.0028) | 922 (-72, 1916) | 0.0021 (-0.0026, 0.0067) | 439679 | 0.04 | 0.039 | 0.04 |
| > 10 minutes | 3220 (531) | -0.0037 (0.0025) | 1795 (605) | -0.0041 (0.0028) | 1425 (-86, 2937) | 0.0004 (-0.0067, 0.0074) | 3989128 | 0.032 | 0.031 | 0.027 |
| ***999 call received to EMS arrival at scene*** |  |  |  |  |  |  |  |  |  |  |
| ≤ 10 minutes | 3676 (544) | 0.0024 (0.0025) | 2203 (548) | 0.0018 (0.0026) | 1473 (925, 2021) | 0.0006 (-0.002, 0.0032) | 2427288 | 0 | 0 | 0 |
| > 10 minutes | 2188 (599) | -0.001 (0.0028) | 1335 (548) | -0.0022 (0.0026) | 853 (-336, 2042) | 0.0012 (-0.0043, 0.0068) | 701635 | 0.086 | 0.084 | 0.086 |
| ***Shockable rhythm*** |  |  |  |  |  |  |  |  |  |  |
| No | 3435 (548) | 0.0025 (0.0026) | 2425 (552) | 0.0017 (0.0026) | 1010 (483, 1536) | 0.0008 (-0.0016, 0.0033) | 1201680 | 0 | 0 | 0 |
| Yes | 9166 (684) | 0.0306 (0.0032) | 6620 (552) | 0.0302 (0.0026) | 2546 (1236, 3857) | 0.0005 (-0.0057, 0.0066) | 5282677 | 0.001 | 0.001 | 0.001 |
| ***Syringes given (2)*** |  |  |  |  |  |  |  |  |  |  |
| ≤ 2 | 9767 (751) | 0.0238 (0.0035) | 7641 (827) | 0.0319 (0.0039) | 2127 (690, 3564) | -0.008 (-0.0148, -0.0013) | Dominated | 0.001 | 0.001 | 0.001 |
| > 2 | 2031 (637) | -0.0029 (0.003) | 1347 (827) | -0.0032 (0.0039) | 685 (-1432, 2801) | 0.0002 (-0.0097, 0.0102) | 2995310 | 0.265 | 0.265 | 0.269 |
| ***Syringes* given (4)** |  |  |  |  |  |  |  |  |  |  |
| ≤ 4 | 5315 (556) | 0.0081 (0.0026) | 3036 (572) | 0.0068 (0.0027) | 2279 (1582, 2976) | 0.0013 (-0.002, 0.0046) | 1782438 | 0 | 0 | 0 |
| > 4 | 936 (573) | -0.0068 (0.0027) | 686 (572) | -0.0061 (0.0027) | 251 (-920, 1422) | -0.0007 (-0.0062, 0.0048) | Dominated | 0.325 | 0.321 | 0.319 |
| ***Witnessed* by** |  |  |  |  |  |  |  |  |  |  |
| Not witnessed | 3470 (560) | 0.002 (0.0026) | 2409 (568) | 0.0022 (0.0027) | 1061 (299, 1823) | -0.0002 (-0.0038, 0.0034) | Dominated | 0.003 | 0.003 | 0.002 |
| EMS | 4738 (787) | 0.0117 (0.0037) | 3804 (568) | 0.0125 (0.0027) | 934 (-829, 2696) | -0.0009 (-0.0091, 0.0074) | Dominated | 0.155 | 0.153 | 0.153 |
| Bystander | 4762 (572) | 0.0068 (0.0027) | 3189 (568) | 0.0049 (0.0027) | 1573 (308, 2837) | 0.0019 (-0.0041, 0.0078) | 843463 | 0.007 | 0.007 | 0.007 |
| ***Bystander CPR*** |  |  |  |  |  |  |  |  |  |  |
| No | 3325 (582) | 0.0032 (0.0027) | 2411 (545) | 0.0029 (0.0026) | 915 (-219, 2048) | 0.0003 (-0.005, 0.0056) | 2859086 | 0.046 | 0.045 | 0.048 |
| Yes | 4022 (538) | 0.0033 (0.0025) | 2456 (545) | 0.0022 (0.0026) | 1566 (964, 2169) | 0.0011 (-0.0018, 0.0039) | 1475919 | 0 | 0 | 0 |
